# Transcription factor TOX maintains the expression of Mst1 in controlling the early mouse NK cell development

**DOI:** 10.7150/thno.81198

**Published:** 2023-03-27

**Authors:** Liang Luo, Peiran Feng, Quanli Yang, Wenkai Lv, Wanqing Meng, Zhinan Yin, Zhizhong Li, Guodong Sun, Zhongjun Dong, Meixiang Yang

**Affiliations:** 1 The Fifth Affiliated Hospital (Heyuan Shenhe People's Hospital), Jinan University, Heyuan 517000, China; 2 The Biomedical Translational Research Institute, Guangzhou Key Laboratory for Germ-free animals and Microbiota Application, Key Laboratory of Ministry of Education for Viral Pathogenesis & Infection Prevention and Control, School of Medicine, Jinan University, Guangzhou, 510632, China; 3 Guangdong Provincial Key Laboratory of Tumor Interventional Diagnosis and Treatment, Zhuhai Institute of Translational Medicine, Zhuhai People's Hospital Affiliated with Jinan University, Jinan University, Zhuhai, 519000, China; 4 Department of Orthopedics, The First Affiliated Hospital, Jinan University, Guangzhou, 510630, China; 5 The First Affiliated Hospital of Anhui Medical University and Institute for Clinical Immunology, Anhui Medical University, Anhui, 230032, China; 6 School of Medicine and Institute for Immunology, Tsinghua University, Beijing, 100084, China

**Keywords:** TOX, NK cell, Mst1, cell development, immune surveillance

## Abstract

**Rationale:** TOX is a DNA-binding factor required for the development of multiple immune cells and the formation of lymph nodes. However, the temporal regulation mode of TOX on NK cell development and function needs to be further explored.

**Methods:** To investigate the role of TOX in NK cells at distinct developmental phases, we deleted TOX at the hematopoietic stem cell stage (*Vav-Cre*), NK cell precursor (*CD122-Cre*) stage and late NK cell developmental stage (*Ncr1-Cre*), respectively. Flow cytometry was used to detect the development and functional changes of NK cell when deletion of TOX. RNA-seq was used to assess the differences in transcriptional expression profile of WT and TOX-deficient NK cells. Published Chip-seq data was exploited to search for the proteins directly interact with TOX in NK cells.

**Results:** The deficiency of TOX at the hematopoietic stem cell stage severely retarded NK cell development. To a less extent, TOX also played an essential role in the physiological process of NKp cells differentiation into mature NK cells. Furthermore, the deletion of TOX at NKp stage severely impaired the immune surveillance function of NK cells, accompanied by down-regulation of IFN-γ and CD107a expression. However, TOX is dispensable for mature NK cell development and function. Mechanistically, by combining RNA-seq data with published TOX ChIP-seq data, we found that the inactivation of TOX at NKp stage directly repressed the expression of Mst1, an important intermediate kinase in Hippo signaling pathway. Mst1 deficient at NKp stage gained the similar phenotype with *Tox^fl/fl^CD122^Cre^* mice.

**Conclusion:** In our study, we conclude that TOX coordinates the early mouse NK cell development at NKp stage by maintaining the expression of Mst1. Moreover, we clarify the different dependence of the transcription factor TOX in NK cells biology.

## Introduction

Natural killer (NK) cells are critical to innate immunity through their cytolytic activity and ability to eliminate tumor cells and pathogen-infected cells 1. NK cells have become one of the preferred cells for cellular immunotherapy and have been clinically used to treat multiple tumors, including breast cancer, lymphoma, and non-small cell lung cancer 2-4. As we all know, NK cells are usually defined as CD3^-^CD56^+^ cells in humans and CD3^-^NK1.1^+^ or CD3^-^NKp46^+^ cells in mice. Notably, different subpopulations classified on the basis of their expression of CD11b, CD27, KLRG1 and CD226 (DNAM-1) have been described in mice 5, 6, with no equivalence established so far between mouse NK cell subsets and human NK cell subsets. This knowledge gap is a formidable challenge that limits the successful clinical utilization of NK cells. Hence, exploring more important NK development and functional regulatory factors will provide experimental basis for NK cell basic biology and tumor biological therapy. NK cells are derived from hematopoietic stem cells (HSCs) in bone marrow (BM) through the successive development and differentiation of a series of progenitor cells, including common lymphoid progenitor (CLP), NK-committed progenitor (pre-NKp), NK cell progenitor (NKp), immature NK (iNK) and mature NK (mNK) cells 7-9. It is worth noting that NKp cells begin to express CD122 which is the β-chain of the IL-15 receptor. Thus, IL-15 integrate with its receptor signaling controlling the expression of many downstream genes to regulate the fate of NK cells from NKp stage. So far, numerous transcription factors have been proven to be located in the downstream of the IL-15 signal and participate in the developmental process of mouse NK cells, including E4BP4, Eomes, T-bet, etc 10-12. Regardless of the deletion of any transcription factors, there are still more or less residual NK cells *in vivo*, which indicates that there are still many unknown regulatory factors which participating in the occurrence of mouse NK cells. Therefore, the role of other transcription factors involved in mouse NK cell commitment needs to be further explored.

Transcription factor TOX (thymocyte selection-associated high mobility group box protein) was first found to be highly expressed in thymic double-positive (DP) T cells under the action of TCR-mediated calcineurin signaling 13. Prior studies have shown that TOX was not only indispensable for the development of CD4^+^T cell lineage 14, 15 and the homeostasis of T follicular helper (Tfh) cells 16, but also crucial for the formation of the exhausted CD8^+^ T cells in tumor microenvironment or chronic infectious diseases 17-19. However, the role of TOX in innate immune system, especially the biological function of NK cells, no matter in human NK cells or in mouse NK cells, is not extremely clear and needs to be further revealed. Kaye J's group indicated that total knock out of TOX in mice resulted in the dramatically loss of NK cells, and the transition of NKp to iNK was severely blocked 20. In addition, Leung W's group revealed that the knock down of TOX2 inherently hindered early development of NK cells, and the overexpression of T-bet could save the development defect of human NK cells 21. All above reports were from germinal knockout mice or *in vitro* experiments, so more accurate and comprehensive reports are urgently needed.

In this study, we found that the expression level of TOX changed dynamically in different stages of NK cell development, and IL-15 stimulation could significantly increase the expression level of TOX. TOX depletion at HSC stage or NKp cell stage significantly affected NK cell homeostasis, manifested by sharply decreased NK cell pools and altered frequencies of NK cell subsets. Interestingly, TOX deletion at HSC stage resulted in the residual NK cell keeping immature but the expression of TOX at NKp stage could maintain the youthful state of NK cells and regulated its function. However, TOX appeared to be dispensable for late NK cells homeostasis. Mechanically, we performed further bioinformatic analysis by combining our RNA-seq data with published high quality TOX ChIP-seq in CD8^+^ T cells 22 and found that TOX participated in NK cell commitment by directly controlling the expression of Mst1. Above all, our study revealed a transcription factor regulation diagram about which TOX control early NK cell development by directly coordinating the expression of Mst1.

## Results

### Construction of various mouse models with conditional deletion of TOX in NK cells at three developmental stages

**Figure 1:**
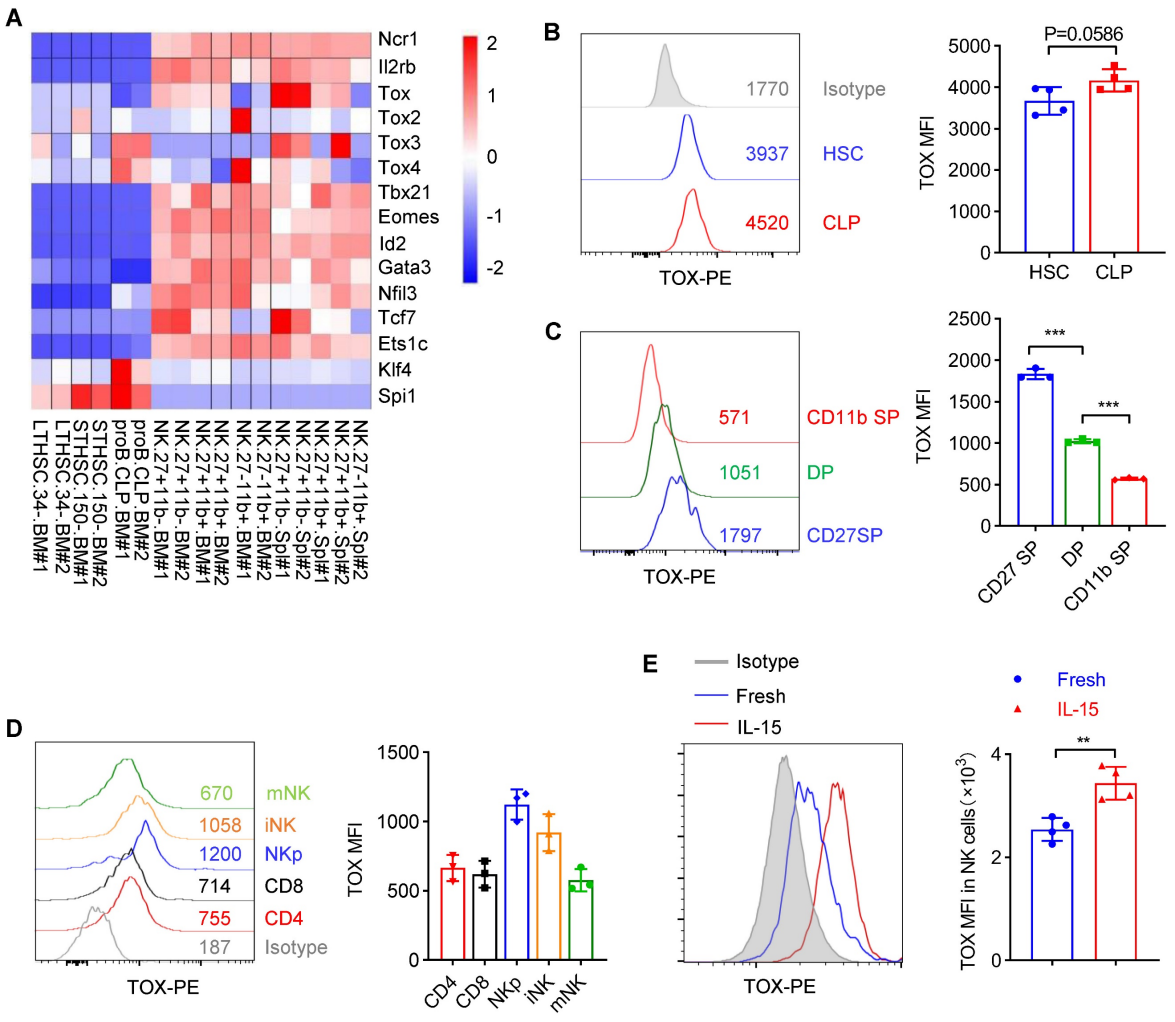
** Dynamic expression of TOX in NK cell subsets. (A)** Heatmap showing the mRNA level of *Tox* and several NK signature genes in various cell subsets by published RNA-seq data (GSE109125). **(B, C)** Representative flow cytometry plot (left) and the mean fluorescent intensity (MFI) analysis (right) of TOX expression in HSC and CLP cells **(B)** (n = 4) or in NK cell subsets **(C)** (n = 3)**. (D)** Representative flow cytometry plot (left) and MFI analysis (right) of TOX expression in CD4^+^ T cells, CD8^+^ T cells, NKp, iNK and mNK cells in spleen (n = 3). **(E)** Representative flow cytometry plot (left) and MFI analysis (right) of TOX expression in NK cells before and after stimulation with 10 ng/mL IL-15 for 16 h (n = 4). Data are representative of two independent experiments.

To clarify the importance of TOX during NK cell development, we firstly checked the expression of TOX from hematopoietic stem cells (HSC) to distinct NK cell subsets along with developmental stages by analyzing published data (GSE109125) from the ImmGen database. It was worth noting that TOX exhibited dynamic expression and upregulated its expression beyond CLP stage (Figure 1A). In addition, in NK cell subsets of BM and spleen, TOX expression was highest at CD27 SP stage and lowest at CD11b SP stage (Figure 1A). The expression levels of TOX in different NK subsets were further confirmed by flow cytometry, and its expression trend was consistent with the results which we described above, exhibiting highest expression at CD27 SP stage (Figure 1B-C). When compared the expression level of TOX between NK cells with T cells, we found that NK cells, especially NKp cells and iNK cells but not mNK cells, hold higher TOX abundances than CD4^+^ T cells and CD8^+^ T cells at steady stage (Figure 1D). Interestingly, the expression of TOX was highest at NKp stage, and the expression level of TOX gradually decreased with the gradual maturation of NK cells (Figure 1D). To examine whether the expression of TOX in NK cells was regulated by IL-15, we compared the expression abundance of TOX in NK cells before and after IL-15 stimulation. The result showed that IL-15 stimulation significantly increased the expression level of TOX in NK cells (Figure 1E). These results suggested that TOX might play different regulatory roles at different stages of IL-15-mediated NK cell development. Therefore, to verify this idea, we generated three genetic models by crossing* Tox^fl/fl^* mice with *Vav-Cre*, *CD122-Cre* or *Ncr1-Cre* transgenic mice to conditionally ablate TOX at HSC, NKp or iNK stage, respectively (Figure S1A). The ablation efficiencies of all the constructed animal models were verified by qPCR (Figure S1B).

### Genetic ablation of TOX in HSC cells leads to severe NK cell lymphopenia

We firstly examined the effect of TOX on lymphocytes from *Tox^fl/fl^Vav^Cre^* mice which depleted TOX at HSC stage. Just like the phenotype reported by Jonathan K's group, that was *Tox^-/^*^-^ mice lost all peripheral lymph nodes, including the mesenteric lymph nodes 20. We indeed noticed the similar phenotype in *Tox^fl/fl^Vav^Cre^* mice (data not show). We selectively displayed the inguinal lymph nodes in Figure S2A. Besides, the deficiency of TOX at HSC stage impaired CD3^+^ T cell development in thymus and spleen (Figure S2B), characterized by the number of CD4^+^ T cells sharply decreased but the number of CD8^+^ T cells stayed normal (Figure S2C-F). In addition, this developmental block affected all CD4^+^ T lineage cells in *Tox^fl/fl^Vav^Cre^* mice, including the development of NKT and Foxp3^+^ regulatory T cells (Figure S2G-J). Interestingly, the T cell subsets in spleen of *Tox^fl/fl^Vav^Cre^* mice showed a more activated phenotype when compared with those in *Tox^fl/fl^* mice (Figure S3A-D), which might due to a compensatory for the abnormally less T cell pools. These results confirmed and reproduced the phenotypes of some previous related research 14, 20, which indicated that TOX played an essential role in CD4^+^ T cell commitment and homeostasis.

**Figure 2:**
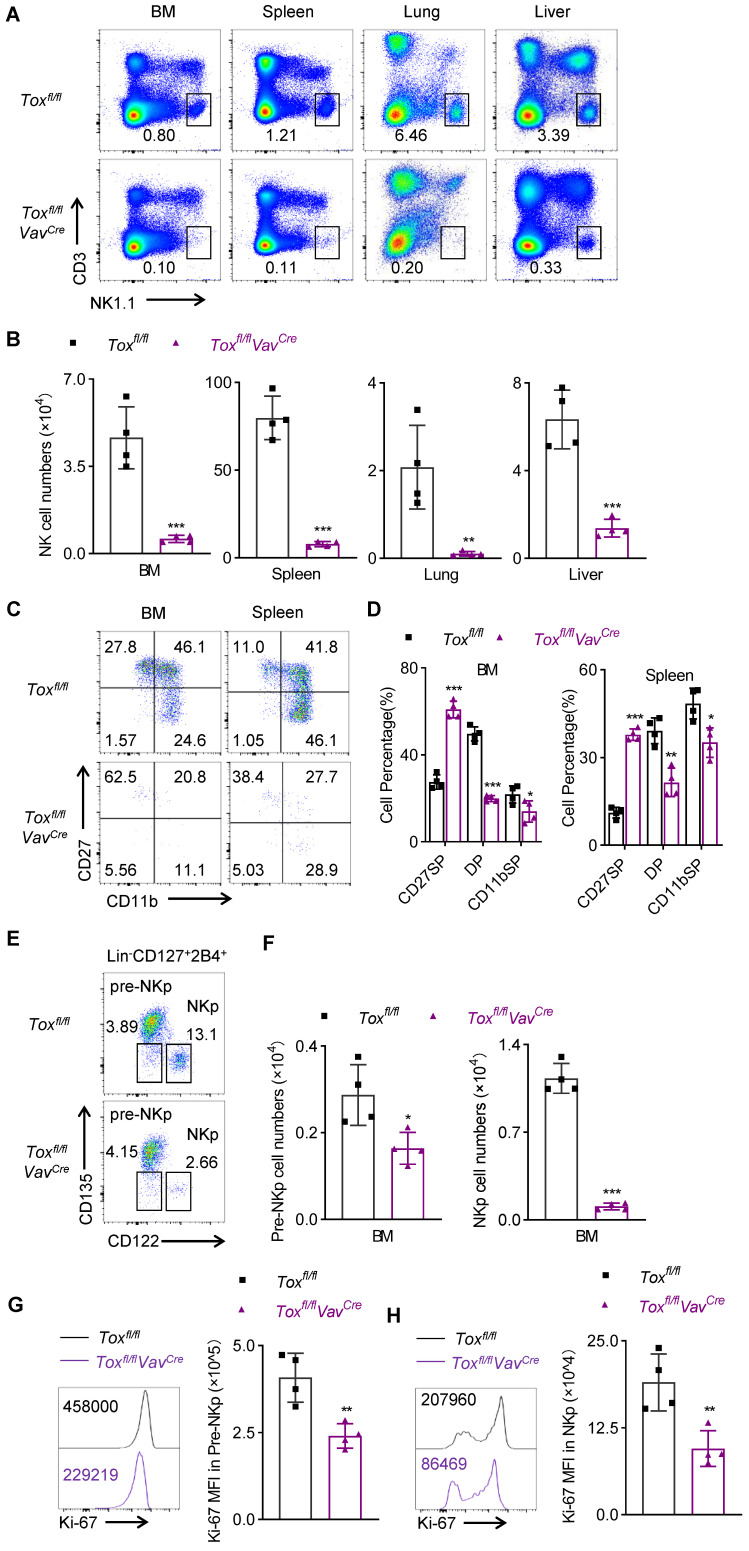
** Genetic ablation of TOX in HSC cells leads to severe NK cell lymphopenia. (A, B)** Representative flow cytometry plot **(A)** and the absolute number **(B)** of NK cells (CD3^-^NK1.1^+^) in BM, spleen, lung and liver of *Tox^fl/fl^* and *Tox^fl/fl^Vav^Cre^* mice (n = 4). **(C, D)** Representative flow cytometry plot **(C)** and the percentage **(D)** of CD27 SP, DP, CD11b SP cells on gated CD3^-^NKp46^+^ cells in BM and spleens of *Tox^fl/fl^* and* Tox^fl/fl^Vav^Cre^* mice (n = 4). **(E, F)** Representative flow cytometry plot **(E)** and the absolute number **(F)** of pre-NKp (Lin^-^CD127^+^2B4^+^CD135^-^CD122^-^) cells and NKp (Lin^-^CD127^+^2B4^+^CD135^-^CD122^+^) cells in BM of *Tox^fl/fl^* and *Tox^fl/fl^Vav^Cre^* mice (n = 4). **(G, H)** Representative flow cytometry plot (left) and the MFI (right) of Ki-67 in pre-NKp cells **(G)** and NKp cells **(H)** in BM of *Tox^fl/fl^* and *Tox^fl/fl^Vav^Cre^* mice (n = 4). Data are representative of two independent experiments.

We further found that the loss of TOX in the hematopoietic stem cells severely disrupted NK cell homeostasis, manifested by a dramatic decrease in the proportion and number of NK cells in multiple organs of *Tox^fl/fl^Vav^Cre^* mice compared with their wild-type littermate controls (Figure 2A-B). To further assess whether the residual NK cells in *Tox^fl/fl^Vav^Cre^* mice were true NK cells or some other cellular lineage bearing a few NK markers, we dissected some classical markers which were expressed by lymphocytes. Undoubtably, the residual NK cells in *Tox^fl/fl^Vav^Cre^* mice didn't express any “non-NK” markers but preserved the classical NK-related markers (Figure S3E). In addition, after NKp cells differentiated into NK cells, three main subsets could be defined according to the different expression of CD27 and CD11b on the surface of NK cells: CD27 SP (CD27^hi^CD11b^lo^); DP (CD27^hi^CD11b^hi^); CD11b SP (CD27^lo^CD11b^hi^). Their differentiation pattern was as follows: CD27 SP→DP→CD11b SP 23. The differences in the proportions of NK cell subsets in the BM and spleen between *Tox^fl/fl^Vav^Cre^* mice and *Tox^fl/fl^* mice were detected by flow cytometry. We noticed that inactivation of TOX at HSC stage resulted in a significant increase in the proportion of immature CD27 SP NK cells and a decline in the percentage of CD11b^+^ mature NK cells compared to the WT controls (Figure 2C-D). To investigate in-depth how TOX orchestrated NK cells development at a spatiotemporal level 24, 25, we further quantified some precursors of NKp cells, including HSCs, CLPs, and CMP cells. The result showed that the numbers of these three populations in *Tox^fl/fl^Vav^Cre^* mice were comparable to those in *Tox^fl/fl^* mice (Figure S4A-B). However, we next noticed that fewer pre-NKp cells and NKp cells remained in BM of *Tox^fl/fl^Vav^Cre^* mice (Figure 2E-F), which suggested an essential role of TOX in early NK cell commitment. Ulteriorly, to further assess the cause of the impaired homeostasis of pre-NKp and NKp cells in *Tox^fl/fl^Vav^Cre^* mice, we detected the proliferation and apoptosis levels in pre-NKp and NKp cells between *Tox^fl/fl^Vav^Cre^* mice and their littermate controls. We found that the capacity of proliferation in pre-NKp and NKp cells from *Tox^fl/fl^Vav^Cre^* mice was comprised, manifested by lower staining of Ki-67 (Figure 2G-H, Figure S4C-D). However, pre-NKp and NKp cells derived from *Tox^fl/fl^Vav^Cre^* mice showed comparable apoptosis level relative to those from wild-type controls (Figure S4E-F). Thus, we concluded that the decreased cell abundance of pre-NKp and NKp cells in *Tox^fl/fl^Vav^Cre^* mice was due to decreased proliferative ability rather than apoptosis levels. These findings collectively indicated that conditional ablation of TOX at HSC stage sorely impaired NK cell development and blocked NK cell commitment.

Next, we sought to determine whether the requirement of TOX for NK cell homeostasis at HSC stage was cell intrinsic. To address this question, we co-transferred BM cells from WT (CD45.1) and *Tox^fl/fl^Vav^Cre^* mice (CD45.2) mice at a 1:1 ratio into CD45.1^+^CD45.2^+^ recipient mice after sublethal irradiation (Fig S5A). After 8 weeks, NK cells derived from TOX-deficient CD45.2^+^ mice were outcompeted by those from WT NK cells in spleen and BM (Figure S5B-D). Moreover, NK cell subset distribution derived from the recipient mice was consistent with that we have described above, that is CD45.2^+^ TOX-deficient NK cells exhibited more immature compared to CD45.1^+^ TOX-sufficient NK cells, as shown by CD11b and CD27 staining (Figure S5E). These findings indicated that TOX regulated NK cell commitment and development in a cell-intrinsic manner at HSC stage.

### The deficiency of TOX at NKp stage impairs NK cell development

**Figure 3:**
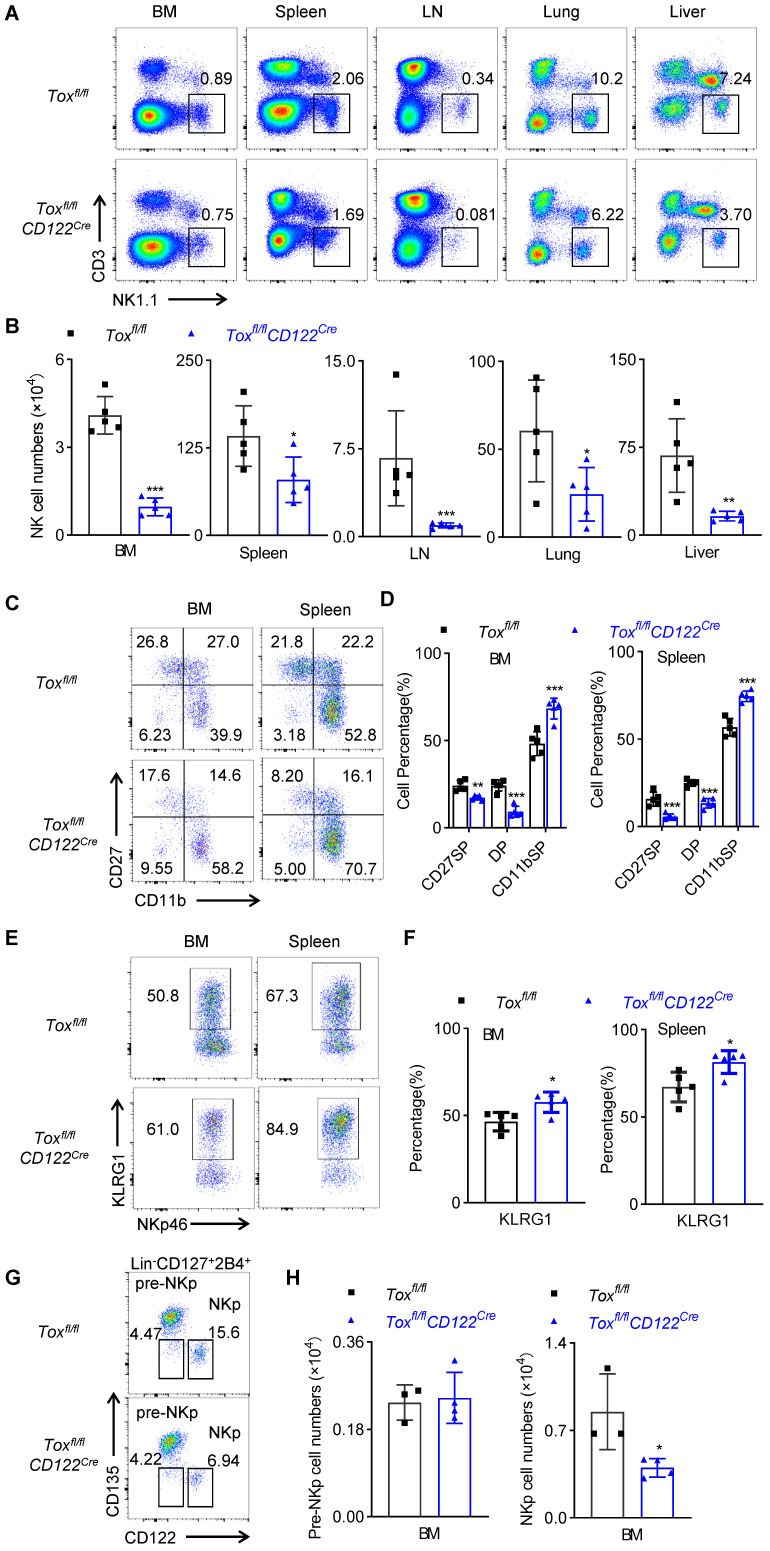
** The deficiency of TOX at NKp stage impairs NK cell homeostasis. (A, B)** Representative flow cytometry plot **(A)** and the absolute number **(B)** of NK cells in BM, spleen, LN, lung and liver of *Tox^fl/fl^* and *Tox^fl/fl^CD122^Cre^* mice (n = 5). **(C, D)** Representative flow cytometry plot **(C)** and the percentage **(D)** of CD27 SP, DP, CD11b SP cells on gated CD3^-^NKp46^+^ in BM and spleen of *Tox^fl/fl^* and *Tox^fl/fl^CD122^Cre^* mice (n = 5). **(E, F)** Representative flow cytometry plots **(E)** and the the percentage **(F)** of KLRG1 expression in BM and spleen of *Tox^fl/fl^* and* Tox^fl/fl^CD122^Cre^* mice (n = 5). **(G, H)** Representative flow cytometry plots **(G)** and the absolute number **(H)** of pre-NKp and NKp cells in BM of *Tox^fl/fl^* and *Tox^fl/fl^CD122^Cre^* mice (n ≥ 3). Data are representative of three independent experiments.

We next detected the proportion and number of NK cells in BM, spleen, lymph nodes, lung and liver of WT mice and *Tox^fl/fl^CD122^Cre^* mice. Similar to *Tox^fl/fl^Vav^Cre^* mice, the deletion of the TOX at NKp stage resulted in a significant reduction in the proportion and absolute number of NK cells in multiple organs (Figure 3A-B). Moreover, the frequencies of CD27 SP, DP NK cell subset were significantly lower but the frequency of CD11b SP NK cell subset was significantly higher in *Tox^fl/fl^CD122^Cre^* mice relative to those in WT mice (Figure 3C-D). In addition, the terminal mature receptor KLRG1 was significantly more abundant on NK cells in *Tox^fl/fl^CD122^Cre^* mice (Figure 3E-F).

These findings suggested that deletion of the TOX at NKp stage promoted the rapid terminal differentiation of NK cells, and the expression of TOX at NKp stage was beneficial to maintain the rejuvenation of NK cells. In fact, we also figured out that *Tox^fl/fl^CD122^Cre^* mice exhibited fewer NKp cells in the BM when compared to those in *Tox^fl/fl^*mice (Figure 3G-H), which indicted that TOX played an indispensable role in the fate decision of NKp cells. Since CD122 was not selectively expressed on NK cells, other immune cells including CD4^+^ T cells also expressed CD122. We further examined the effect of TOX on T cell homeostasis following deletion at the NKp stage. The results showed that the deficiency of TOX at NKp stage did not affect the proportions and numbers of CD3^+^ T cells, CD4^+^ T cells, CD8^+^ T cells and Treg cells in the spleen and lymph nodes (Figure S6A-D). In summary, deletion of TOX at NKp stage severely retarded NK cell development and accelerated NK cell terminal maturation.

### TOX expression at NKp stage plays a cell-intrinsic role in NK cell development

Owing to the *Tox^fl/fl^CD122^Cre^* mice are not NK cell-specific knockout models, the above results may not be caused by the inactivation of TOX in NK cells. To exclude this possibility, bone marrow chimera assay was designed (Figure S7A). After 8 weeks of BM hematopoietic reconstruction, the development of NK cells in spleen and BM of recipient mice was detected by flow cytometry (Figure S7B). The results showed that the proportions and absolute numbers of NK cells in the spleen and bone marrow of recipient mice derived from *Tox^fl/fl^CD122^Cre^* mice were significantly lower than those derived from the control mice (Figure S7C-D). To corroborate the cell-intrinsic function of TOX in the NK cell differentiation, we dissected NK cell subset distribution between CD45.2^+^ NK cells and CD45.1^+^ NK cells in the same recipient mice. Consistently, CD45.2^+^ NK cells preserved higher frequency of CD11b SP NK cell subset and lower frequencies of CD27 SP and DP NK cell subsets (Figure S7E-F). These results indicated that the regulation of TOX on the development and differentiation of NK cells was cell-intrinsic.

### TOX is dispensable for the homeostasis of immature NK cells

**Figure 4:**
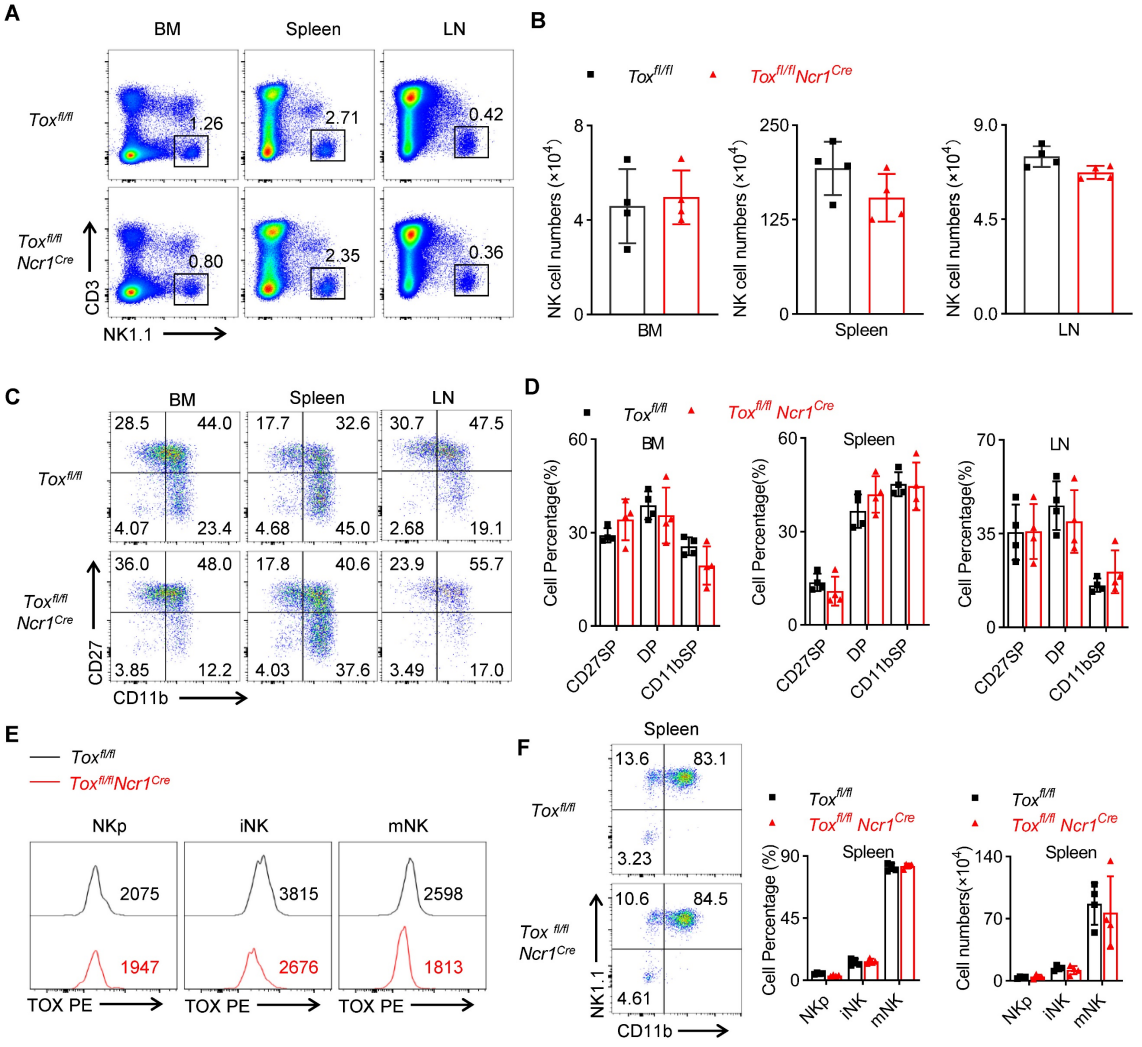
** TOX-deletion in *Ncr1^+^* NK cells does not influence NK cell development. (A, B)** Representative flow cytometry plot **(A)** and the absolute number **(B)** of NK cells in BM, spleens, LN of *Tox^fl/fl^* and *Tox^fl/fl^Ncr1^Cre^* mice (n = 4). **(C, D)** Representative flow cytometry plot **(C)** and the percentage **(D)** of CD27 SP , DP, CD11b SP cells on NK cells in BM, spleen and LN of *Tox^fl/fl^* and *Tox^fl/fl^Ncr1^Cre^* mice (n = 4). **(E)** Comparison of the expression level of TOX in NKp , iNK and mNK cells in spleen of *Tox^fl/fl^* mice and *Tox^fl/fl^Ncr1^Cre^* mice (n = 4). **(F)** The representative flow cytometry plot (left), the percentage (middle) and the number (right) of NKp, iNK and mNK cells in spleen from *Tox^fl/fl^* mice and *Tox^fl/fl^Ncr1^Cre^* mice (n = 4). Data are representative of three independent experiments.

The preceding results demonstrated that TOX was critical in IL-15-mediated early development of NK cells, but the role of TOX in iNK cell homeostasis remained unclear. To clarify this question, we compared the proportion and number of NK cells in BM, spleen and lymph nodes between *Tox^fl/fl^Ncr1^Cre^* mice and the control mice. Differently, we found that *Tox^fl/fl^Ncr1^Cre^* mice contained comparable NK cells with WT mice in the detected organs (Figure 4A-B). Moreover, there was no significant difference in the proportion of each subset between TOX-deficient NK cells and WT NK cells as shown by CD11b and CD27 staining (Figure 4C-D). To avoid the in-efficient deletion mediated by *Ncr1-Cre*, we further compared the expression level of TOX in iNK and mNK cells from *Tox^fl/fl^Ncr1^Cre^* mice and the controls. The results showed that the expression of TOX in iNK and mNK cells but not in NKp cells remarkably reduced in *Tox^fl/fl^Ncr1^Cre^* mice compared to those in *Tox^fl/fl^* mice (Figure 4E), which consistent with that the *Ncr1-Cre* mediated deletion of *Tox* from iNK stage. Also, the ablation of TOX using *Ncr1-Cre* didn't affect iNK and mNK cell percentage and absolute number when compared to WT controls (Figure 4F). Together, these results suggested that *Ncr1-Cre* mediated deletion of TOX was indeed dispensable for iNK development.

### Deletion of TOX at NKp stage impairs NK cell function

**Figure 5:**
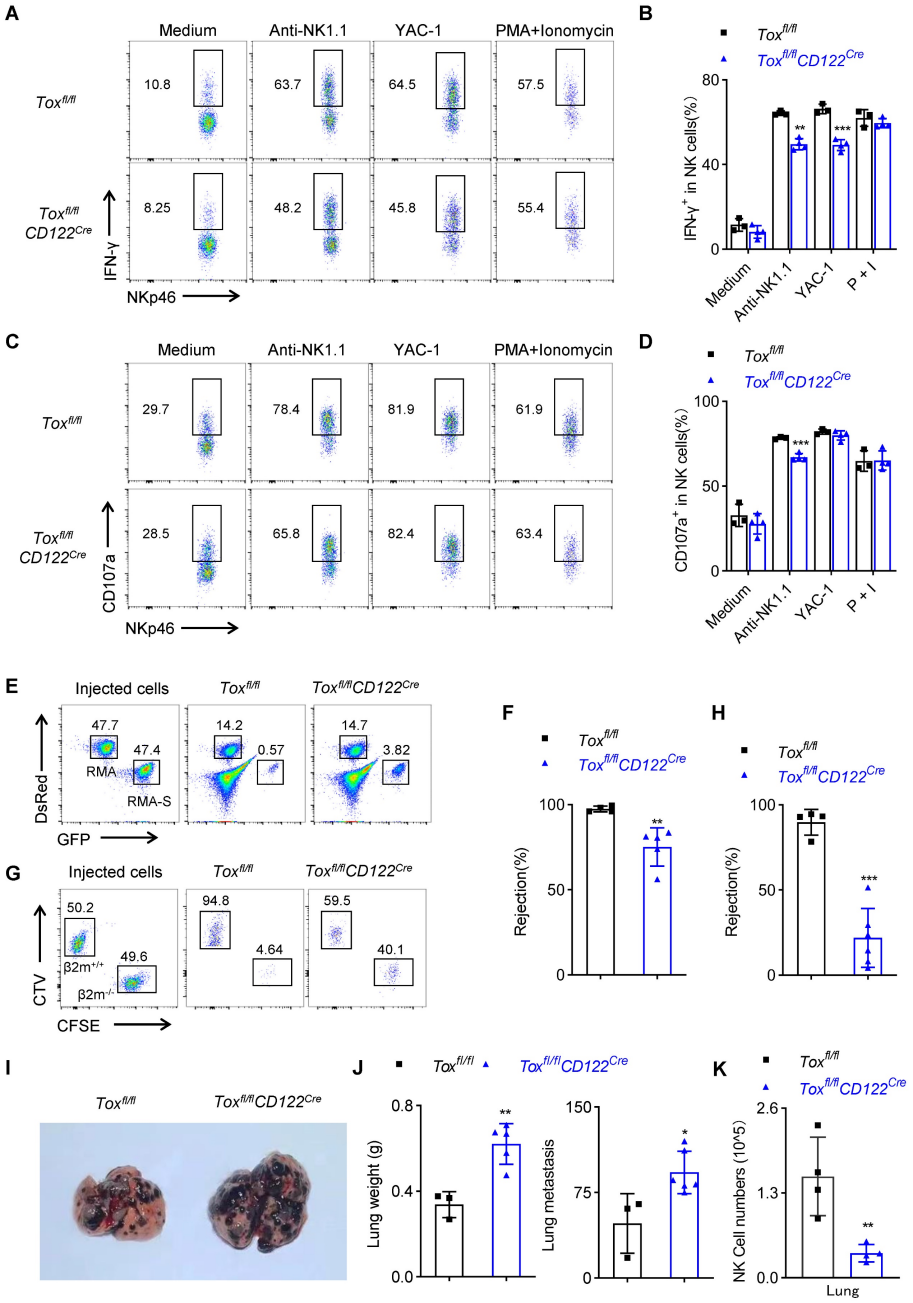
** TOX deficiency at NKp stage impairs NK cell function *in vitro* and *in vivo*. (A-D)** Representative flow cytometry plot **(A, C)** and the percentage **(B, D)** of IFN-γ^+^** (A, B)** or CD107a^+^ **(C, D)** NK cells in spleen of *Tox^fl/fl^* and *Tox^fl/fl^CD122^Cre^* mice (n ≥ 3). **(E, F)** Representative flow cytometry plot **(E)** and the percentage **(F)** of rejected RMA-S cells of *Tox^fl/fl^* and *Tox^fl/fl^CD122^Cre^* mice (n ≥ 4). **(G, H)** Representative flow cytometry plot **(G)** the percentage **(H)** of rejected *β2m^-/-^* splenocytes cells from the spleen of *Tox^fl/fl^* and *Tox^fl/fl^CD122^Cre^* mice (n ≥ 4). **(I, J)** Representative plot **(I)**, the lung weights (J, left) and numbers (J, right) of tumor nodules in *Tox^fl/fl^* and *Tox^fl/fl^CD122^Cre^* mice (n ≥ 4). **(K)** The number of NK cells in the lung from *Tox^fl/fl^* mice and *Tox^fl/fl^CD122^Cre^* mice 14 days after intravenous injection of 2 × 10^5^ B16/F10 cells (n = 4). Data are representative of at least two independent experiments.

NK cells play a central role in rejecting allogeneic bone marrow cells and tumors. We first measured the ability of NK cells function *ex vivo*. The results showed that following the stimulation by plate-coated antibodie against ITAM-containing receptor NK1.1 or with NK cell-sensitive YAC-1 cell lines, the spleen NK cells from *Tox^fl/fl^CD122^Cre^* mice produced less IFN-γ compared with those from WT mice (Figure 5A-B). At the same time, the residual spleen NK cells from *Tox^fl/fl^CD122^Cre^* mice exhibited a defect in CD107a expression under stimulation by anti-NK1.1 (Figure 5C-D). Moreover, an extensive *in vivo* analysis showed that TOX-deleted mice couldn't effectively rejected the mismatched β2m^-/-^ splenocytes and killed RMA-S cells, when compared with the control mice (Figure 5E-H). In addition, we established a mouse B16 melanoma lung metastasis model in two groups of mice. *Tox^fl/fl^CD122^Cre^* mice exhibited more severe pulmonary melanoma burden compared with WT mice (Figure 5I-J). We further dissected the immune cells in the lungs and found *Tox^fl/fl^CD122^Cre^* mice indeed possessed fewer NK cells in tumor microenvironment relative to *Tox^fl/fl^* mice (Figure 5K). Hence, we concluded that the worse capacity of antimetastatic phenotype in *Tox^fl/fl^CD122^Cre^* mice was likely owing to the dramatical deficiency of NK cells in the lungs. In general, the above results showed that deletion of TOX at NKp stage compromised NK cell immunosurveillance.

### TOX is dispensable for terminal NK cell function

**Figure 6:**
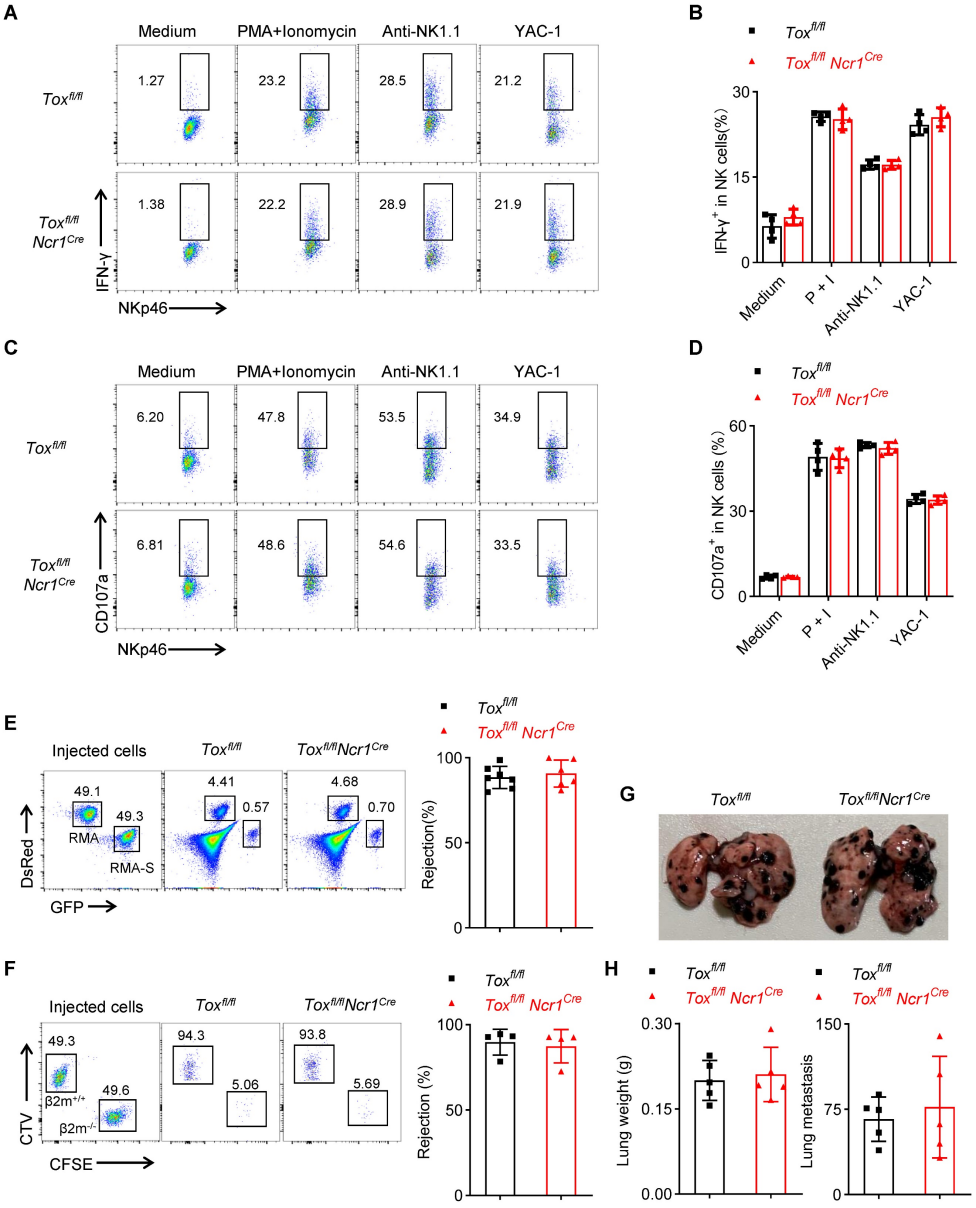
** Tox is not required for terminal NK cell function. (A-D)** Representative flow cytometry plot **(A, C)** and the percentage **(B, D)** of IFN-γ^+^** (A, B)** or CD107a^+^ **(C, D)** NK cells in the spleen of *Tox^fl/fl^* and *Tox^fl/fl^Ncr1^Cre^* mice (n = 4). **(E, F)** Representative flow cytometry plot (left) and the percentage (right) of rejected RMA-S cells **(E)** or *β2m^-/-^* splenocytes **(F)** of *Tox^fl/fl^* and *Tox^fl/fl^Ncr1^Cre^* mice (n ≥ 4). **(G, H)** Representative plot **(G)**, the lung weight **(H**, left**)** and number of tumor nodules **(H**, right**)** in *Tox^fl/fl^* and *Tox^fl/fl^CD122^Cre^* mice (n = 5). Data are representative of at least two independent experiments.

The above results confirmed that the deletion of TOX in late NK cells did not affect the development of NK cells in the spleen and BM, but whether it affected NK cell function remains to be questioned. We found that NK cells from *Tox^fl/fl^Ncr1^Cre^* mice retained comparable IFN-γ expression and CD107a production when compared to those from WT controls (Figure 6A-D). At the same time, just like the control mice, *Tox^fl/fl^Ncr1^Cre^* mice also successfully eliminated the mismatched β2m^-/-^ splenocytes and removed NK-sensitive RMA-S cells (Figure 6E-F). In addition, the lung metastasis of B16 cells in *Tox^fl/fl^Ncr1^Cre^* mice was comparable with the control mice, as evidenced by the equal increases in the lung weights and approximate numbers of tumor colonies between *Tox^fl/fl^Ncr1^Cre^* mice and *Tox^fl/fl^* mice (Figure 6G-H). In summary, the ablation of TOX at iNK stage didn't affect NK cell function no matter *in vivo* or *in vitro*.

### TOX participates in the early development of NK cells by regulating the expression of Mst1

**Figure 7:**
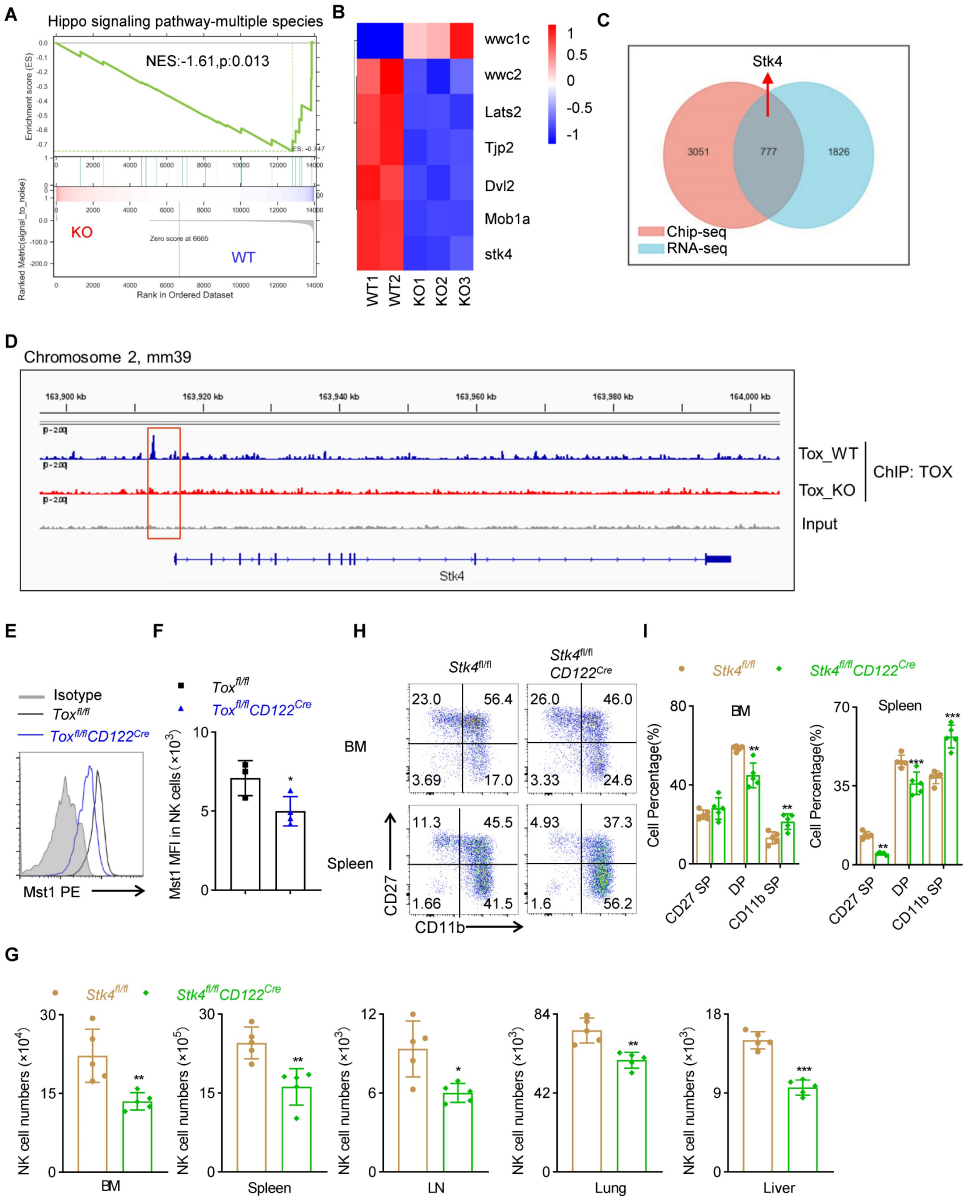
** TOX orchestrates NK cell homeostasis by controlling Mst1 expression. (A)** Pathways commonly enriched in WT and TOX-deficient NK cells based on GSEA analysis of RNA-seq datasets. **(B)** Heatmap of differentially expressed genes involved in Hippo signaling pathway in NK cells from *Tox^fl/fl^* and *Tox^fl/fl^CD122^Cre^* mice. **(C)** Venn diagram showing shared genes in online published Chip-seq dataset (GSE93953) with our RNA-seq datasets. **(D)** The Integrated Genome Viewer (IGV) screenshots exhibiting ChIP-seq profiles in *Stk4* loci. **(E, F)** Representative flow cytometry plot** (E)** and the MFI of Mst1 **(F)** in spleen NK cells of *Tox^fl/fl^* and *Tox^fl/fl^CD122^Cre^* mice (n ≥ 3). **(G)** The absolute number of NK cells in BM, spleen, LN, lung and liver of *Stk4^fl/fl^* and* Stk4^fl/fl^CD122^Cre^* mice (n = 5). **(H, I)** Representative flow cytometry plot **(H)** and the percentage **(I)** of CD27 SP, DP, CD11b SP cells on gated CD3^-^NKp46^+^ cells in BM and spleen of *Stk4^fl/fl^* and* Stk4^fl/fl^CD122^Cre^* mice (n = 4). Data (E-I) are representative of at least two independent experiments.

In order to reveal the molecular mechanism by which TOX affected the development and function of NK cells at NKp phase, we first performed global RNA sequencing (RNA-seq) of sorted NK cells form spleen between *Tox^fl/fl^CD122^Cre^* mice and *Tox^fl/fl^* mice. We found that 2603 down-regulated genes and 929 up-regulated genes with a fold change greater than 2 in NK cells of *Tox^fl/fl^CD122^Cre^* mice compared with WT controls (Figure S8A-B). To further clarify how TOX regulated NK cell homeostasis, we performed GSEA analysis on RNA-seq datasets containing WT and TOX-deficient NK cells. It was worth noting that one gene set, “Hippo signaling pathway”, was enriched (Figure 7A). Indeed, some important genes involved in Hippo signaling pathway were significantly down-regulated in TOX-deficient NK cells when compared with WT NK cells, such as *Stk4* (which encoding the protein Mst1), *Lats2*, *Mob1a* and so on (Figure 7B). NK cells are regarded as the innate counterpart to CD8^+^ T cells, and there are many analogous processes in NK cell and CD8^+^ T cell development. In fact, there are a series of transcription factors governing early developmental programs and activation of NK cells parallel to those of CD8^+^ T cells 26, 27. Thus, to further interrogate how TOX modulated the key regulators in early NK cell development, we constructed a TOX-dependent transcriptional network by combining analysis of existing ChIP-seq data of CD8^+^ T cells with our RNA-seq data. A Venn diagram analysis revealed that 777 of different expressed genes (DEG) were directly bound by TOX (Figure 7C). These 777 genes were either up- or down-regulated in our RNA-seq, suggesting that TOX could exert as trans-activator and repressor. Among those genes, we identified a TOX binding site, which located at the 2.5 kb upstream of the promoter of the gene* Stk4* (Figure 7D). Evidently, the expression level of Mst1 (encoded by *Stk4*) was indeed lower in NK cells of *Tox^fl/fl^CD122^Cre^* mice than that in control mice (Figure 7E-F, S8C). In addition, similar to TOX, short-term IL-15 stimulation drove the expression of Mst1in NK cells (Figure S8D). To confirm that TOX participated in NK cell development by controlling the expression of Mst1, we constructed conditional knockout mice which were deleted Mst1 at NKp stage (Figure S8E-F). The result showed that *Stk4^fl/fl^CD122^Cre^* mice contained fewer NK cells when compared to *Stk4^fl/fl^* mice in the detected tissues and organs (Figure 7G, S8G). At the same time, comparing to the control mice, *Stk4^fl/fl^CD122^Cre^* mice exhibited lower frequencies of CD27 SP, DP NK cell subsets but higher frequency of CD11b SP NK cell subset (Figure 7H-I). These phenotypes were coincided with *Tox^fl/fl^CD122^Cre^* mice. Taken together, these results indicated that TOX participated in early development of NK cell likely to directly regulate the expression of Mst1.

## Discussion

Although TOX is predominantly known for its critical functions in T cells, its biological functions extend to other immune cells, such as ILCs and NK cells 28. The importance of TOX in NK cell was initially recognized in germinal knockout mice or *in vitro* experiments, and the role of TOX in NK cell development and functional maturation cannot be fully elucidated. In this study, we found that TOX played different roles in distinct developmental stages of NK cells, characterized by the fact that TOX was critical for early NK cell development and function, but was dispensable for late NK cell homeostasis and function.

Although NK cells are members of the innate immune system, their powerful ability to clear virus-infected cells and malignantly transformed tumor cells makes them popular 29, 30. The homeostasis and function of NK cells depend on the precise regulation of many intracellular and extracellular biochemical signals, among which transcription factors are indispensable for the regulation of NK cell differentiation and maintenance 31. In recent years, the role of transcription factor TOX in the production of immune cells, especially exhausted T cells, has attracted great attention from researchers, and important breakthroughs have been made. However, the regulatory effect of TOX on NK cells and its mechanism are still exceedingly unclear. Only in 2010, Kaye J's group reported that TOX was highly expressed in iNK cells and mNK cells in BM, and found that systemic deletion of TOX in mice reduced the number of NK cells in spleen and BM 20. In addition, in 2011, Choi I's work confirmed that TOX2 was a key regulator in the process of differentiation from human cord blood stem cells to NK cells through gene silencing and overexpression, but the overexpression of TOX2 in NK cells does not affect the function of NK cells 32. The existing reports were derived from germline knockout mice or *in vitro* experiments, and the experimental results cannot accurately reflect the regulation of TOX on NK cell function. Therefore, it is necessary to use some new stage-specific animal models to clearly explore the specific regulatory effects of TOX on the development and function of NK cells and the related mechanism.

In our research, *Tox^fl/fl^* mice were mated with *Vav-Cre*, *CD122-Cre*, or *Ncr1-Cre* mice to inactivate TOX conditionally at HSC, NKp or iNK stage, respectively. Through a variety of conditional knockout animal models, it was expected to clarify the temporal regulation mode of TOX on NK cell development and function. Consistently, there was no doubt that *Tox^fl/fl^Vav^Cre^* mice could not find any lymph nodes and held fewer T cells, especially CD4^+^ T cells, including Treg cells and NKT cells. Next, we found that both deletion of TOX at HSC stage and the clearance of TOX in NK cell precursors severely blocked NK cell development. At the same time, compared to WT NK cells, NK cells from *Tox^fl/fl^Vav^Cre^* mice appeared to stay immature. Unlikely, *Tox^fl/fl^CD122^Cre^* mice show higher frequency of CD11b SP NK cell subset, lower proportions of CD27 SP NK cell subset and DP NK cell subset compared to the wildtype controls. This means that the expression of TOX at NKp stage of NK cells can maintain the youthful state of NK cells and prevent NK cell differentiation towards terminal maturation. The NK subset distribution differences between *Tox^fl/fl^Vav^Cre^* mice and *Tox^fl/fl^CD122^Cre^* mice might be due to that TOX expression at different developmental stages of NK cells can regulate different developmental molecules. Interestingly, TOX ablation in mature NK cells does not affect NK cell homeostasis. These results indicate that TOX plays an integral role during early development of NK cells, whereas late NK cell homeostasis is independent of TOX.

Up to now, the effect of TOX on NK cell function has not been reported. To clarify this problem, we conducted a series of classic NK functional experiments *in vivo* and *in vitro*. The results prompted that TOX played an essential role in NK-mediated immunosurveillance at the early stage, characterized by altered IFN-γ and CD107a expression, lower rejection to NK-sensitive target cells, and impaired antimetastatic function in *Tox^fl/fl^CD122^Cre^* mice. However, the ablation of TOX at late stage didn't influence NK cell function. These results indicate TOX is more crucial for the functional integrity of early NK cells but not for that of late NK cells.

To inspect how TOX orchestrated NK cell homeostasis at NKp stage, we first performed RNA-seq using sorted NK cells in spleen of *Tox^fl/fl^CD122^Cre^* mice and WT controls. Our RNA-seq data revealed that TOX deficiency at NKp stage brought out significant changes in genome of NK cells of *Tox^fl/fl^CD122^Cre^* mice compared to that of WT mice. In addition, some important genes which involved in Hippo signaling pathway tremendously down-regulated in NK cells of *Tox^fl/fl^CD122^Cre^* mice, including *stk4*, which encoded the Mst1 protein kinase. Since transcription factor control gene expression by activating or inhibiting gene transcription, so we conducted a transcript regulation plot by combing our RNA-seq data with online Chip-seq data in CD8^+^ T cells 22. We found 777 different expression genes could directly bind by TOX. Interestingly, *Stk4* was one of the 777 TOX-binding DEGs. The expression of Mst1 was indeed lower in NK cells of *Tox^fl/fl^CD122^Cre^* mice than that in WT controls and its expression was driven by IL-15 signal in NK cells. What role of Mst1 plays in NK cells? To reveal this query, we generated *Stk4^fl/fl^CD122^Cre^* mice, which deleted Mst1 at NKp stage. Strikingly, the ablation of Mst1 at NKp stage gravely compromised NK cell homeostasis, manifested by alter NK cell numbers, more frequency of terminal mature CD11b SP NK cell subset but less frequencies of CD27 SP and DP NK cell subset. The phenotype of *Tox^fl/fl^CD122^Cre^* mice was similar to *Stk4^fl/fl^CD122^Cre^* mice. Thus, we inferred that TOX participated in NK cell development likely by directly controlling the expression of Mst1.

In summary, our study indicates that TOX is essential for NK cell commitment, differentiation, maturation and function. In addition, TOX regulates NK cell homeostasis by regulating the expression of the key kinase Mst1. The aim of this study is to enrich the regulatory network of transcription factors in the developmental map of NK cells, and to provide scientific and feasible theoretical basis and factual support for the use of NK cells in clinical research and therapy.

## Materials and methods

### Mice

*CD122-Cre* and *Ncr1-Cre* mice were generated in our lab. *Vav-Cre* mice. *Stk4^fl/fl^* mice, *β2m*-deficient mice and CD45.1 mice were purchased from the Jackson Laboratory. *Tox^fl/fl^* mice were kindly given by Dr. Lilin Ye (Third Military Medical University, Chongqing, China). All mice used in this study were between 6 and 10 weeks of age on a C57BL/6J background and were bred and housed in the specific pathogen-free animal facilities of Jinan University under controlled temperature (22 ±1 °C) and exposed to a constant 12-hour light/dark cycle. Mice used in our study were genders and ages matched. Male mice and female occupy half of total animals, respectively. The experimental operations and related procedures involved were approved by the Animal Ethics Committee of Jinan University (The institutional animal ethics clearance number: IACUC-20211202-01).

### Flow cytometry

All flow cytometry results were obtained on a BD FACS Verse™ (three-laser flow cytometry analyzer, BD Biosciences) or Cytek® Aurora (3 Laser 16V-14B-8R, Cytek). Monoclonal antibodies against mouse FITC /PE/PE-Cy7/APC/APC-Cy7/Percp/eFluor 450 conjugated anti-CD3(145-2C11), FITC/APC/APC-Cy7/PE-Cy7 conjugated anti-CD4(GK1.5), FITC/PE/APC/APC-Cy7 conjugated anti-CD8 (53-6.7), PE/APC conjugated anti-NK1.1(PK136), FITC/PE/PE-Cy7/APC conjugated anti-NKp46 (29A1.4), eFluor 450/PE-Cy7/APC/Percp conjugated anti-CD11b (M1/70), PE-Cy7/APC-Cy7 conjugated anti-CD44 (1M7), APC/Super Bright 436 conjugated anti-CD62L (MEL-14), PE/eFluor 450 conjugated anti-Foxp3 (FJK-16s), FITC/Percp/eFluor 450 conjugated anti-CD122 (TM-b1), PE conjugated anti-CD135 (A2F10), Percp conjugated anti-Sca-1 (D7), APC conjugated anti-2B4 (244F4), PE conjugated anti-TOX (TXRX10), PE-Cy7/APC conjugated anti-KLRG1 (2F1), eFluor 450/APC-Cy7 conjugated anti-CD117 (2B8), PE/PE-Cy7 conjugated anti-CD127 (SB/199), eFluor 450/ APC conjugated anti-CD107a (1D4B), PE conjugated F(ab) Donkey Anti Rabbit IgG (12-4739-81) and isotype controls were purchased from eBioscience (San Diego, CA). Polyclonal antibody against mouse MST1 (Cat#3682T) was purchased from Cell Signaling Technology (Beverly, MA). Cell Tracker™ Violet (Cat#C10094) and Foxp3/transcription factor staining buffer kit (Cat#00-5523-00) were purchased from eBioscience (San Diego, CA). Monoclonal antibodies against mouse PE-Cy7/PE conjugated anti-IFN-γ (XMG1.2), PE-Cy7/PE conjugated anti-CD27 (LG.3A10), Percp conjugated anti-TNF-α (MP6-XT22) and anti-Lineage Cocktail (Cat#133310) were purchased from Biolegend (San Diego, CA). Fixation/Permeabilization Solution Kit (555028) was purchased from BD Biosciences (Mississauga, Ontario, Canada). Dead cells were ruled out from analysis by using LIVE/DEAD fixable Aqua dead cell stain kit (Cat#L34965, Invitrogen) according to the manufacturer's instructions. Flow cytometry data was analyzed using Flowjo software.

### RNA isolation and quantitative reverse transcription-PCR

Total RNA was obtained from purified indicated cells of various mice and reverse-transcribed with a Takara reverse transcription kit. The primers for the mouse genes were: *Tox*-F: TCCTCGCACAGAGATCAACT, *Tox*-R: TGTCTTGTGGCGCTGCTC. *Stk4*-F: ATATCATTCGGCTACGGAAC, *Stk4*-R: GCCTTGATATCTCGGTGTAT. The primers for internal control gene *Gapdh* were: *Gapdh*-F: CCAGCTTAGGTTCATCAGGT, *Gapdh*-R: TTGATGGCAACAATCTCCAC. Real-time PCR was performed on the BioRad CFX Connect cycler.

### Detection the membrane expression of CD107a and the secretion of intracellular IFN-γ

Mice were treated with intraperitoneal injection of poly I:C (150 µg) for 16 h. Poly I: C-activated splenocytes (2×10^6^) were co-cultured with the same number of different target cells. For anti-NK1.1 stimulation, 24-well plates were pre-coated with 5 ug/ml anti-NK1.1 (PK136) antibodies in a 4 ℃ refrigerator overnight. BD GolgiStop™ reagent (BD Biosciences) was added to all culture systems to inhibit the transport of endocrine proteins out of cells. At the same time, APC-conjugated anti-CD107a antibody or respective control isotype were added at the beginning of incubation. In order to ensure that the total amount of cells and the final volume of all groups are consistent, for tumor cell stimulation, YAC-1 cells were put together with splenocytes at 1:1 ratio. Splenocytes stimulated with PMA (50 ng/mL) plus ionomycin (1 µM) were used as a positive control. Medium only was used as a negative control. 4-6 h after stimulation, cell mixtures were harvested for detection the membrane expression of CD107a and the secretion of intracellular IFN-γ.

### *In vivo* splenocyte rejection assay

Mice were treated with intraperitoneal injection of poly I:C (150 µg) for 16 h. On the day of the experiment, spleen cells from wild-type mice and *β2m^-/-^* mice were labeled with 5 μg/mL Cell Tracker™ Violet (CTV) and 1 μM CFSE, respectively. The two cells were then intravenously injected at 1:1 ratio into the indicated mice which were pretreated with poly I:C 16 h before. 5 h later, CFSE-positive cells from the spleens were determined by flow cytometry.

### *In vivo* RMA-S clearance assay

Mice were treated with intraperitoneal injection of poly I:C (150 µg) for 16 h. On the day of the experiment, the pre-cultured NK cell-sensitive lymphoma cells (RMA-S) expressing green fluorescent protein GFP and non-NK cell-sensitive lymphoma cells (RMA) expressing red fluorescent protein Ds-Red were collected. The two cells were adjusted to the same concentration. RMA-S and RMA cells were mixed at a 1:1ratio, then the mixture was injected intraperitoneally (2×10^6^/mouse) into the indicated mice which were pretreated with Poly I:C 16 h before. After 16 hours, the mice were sacrificed by cervical dislocation, then the RMA-S and RMA cells in the abdominal cavity of the indicated mice were collected to calculate the clearance ratio of RMA-S by flow cytometry.

### B16 melanoma lung metastasis mouse model

B16/F10 mouse melanoma cells in logarithmic growth phase were resuspended with 1×Hank's balance solution (HBSS). Then, the cells were injected into the indicated mice (2×10^5^cells/mouse) by tail vein infusion. After 14 days, the mice were sacrificed. The lungs were photographed and weighed. At the same time, the number of melanoma nodules on the lung surface was counted under a dissecting microscope.

### Adoptive transfer assay

Donor wild-type mice (CD45.1^+^) and conditional knockout mice (CD45.2^+^) were injected 300μl 10 mg/mL 5-FU (for a 20g adult mice) via the tail vein respectively to mobilize hematopoietic stem cells. Five days later, the mice were sacrificed. The bone marrow cells of the CD45.1^+^ mice and the CD45.2^+^ mice were mixed at 1:1 ratio and injected into the CD45.1^+^CD45.2^+^ mice after sublethal irradiation through the tail vein. After about 6-8 weeks of bone marrow cell reconstruction, the development of NK cells derived from gene-deleted mice (CD45.2^+^) and from WT mice (CD45.1^+^) were detected. During the process of bone marrow cell reconstruction, mice were fed antibiotic water (Neomycin,1mg/ml) once or twice a week.

### RNA-seq

NK cells were sorted from the spleen of both *Tox^fl/fl^* and *Tox^fl/fl^CD122^Cre^* mice. Total RNA from NK cells was extracted using RNeasy Micro Kit (50) (Qiagen 74004) according to manufacturer's protocol. The extracted RNA was evaluated for purity and quality using a NanoDrop 2000 spectrophotometer (Thermo Scientific, USA). The integrity of the extracted RNA was assessed using an Agilent 2100 Bioanalyzer (Agilent Technologies, Santa Clara, CA, USA). Then the gene libraries were constructed according to the manufacturer's instructions using Single Cell Full Length mRNA-Amplification Kit (Vazyme, N712-03, Nanjing, China) and TruePrep DNA Library Prep Kit V2 for Illumina (Vazyme, TD502-02, Nanjing, China). Transcriptome sequencing and subsequent data analysis were performed at OE Biotech Co., Ltd. (Shanghai, China).

### ChIP-Seq Data Analysis

ChIP-seq of CNS-infiltrating CD8^+^ T cells was originated from the Gene Expression Omnibus with accession no. GSE93953. Quality assessment of raw reads was estimated by FastQC (0.11.9) and adaptors were purged using Trim galore (0.6.3). The processed data were mapped to the mouse reference genome (MM10), which was downloaded from the UCSC repository using Bowtie (2.1.0). The aligned results transformed into bigWig format by Bedtools (2.26.0) were visualized on the Integrated Genome Viewer from the Broad Institute. MACS (2.1.1) was utilized to call binding sites (peaks) relative to Input libraries with the p-value threshold set as 1 × 10^-4^. Finally, called peaks were noted against the mouse reference genomes (mm10) in HOMER.

### Statistics

The data presented above was shown as mean ± SEM. Two-tailed Student's t test was used to analyze the differences between the two groups of data. For the comparison of differences between paired samples, paired Student's t test was used for analysis. The following terminology is used to show statistical significance: *P<0.05, **P<0.01, ***P<0.001. All data analysis was performed by GraphPad Prism 8 software.

## Supplementary Material

Supplementary figures.
