# Supplementary material for: Transcription factor TOX maintains the expression of Mst1 in controlling the early mouse NK cell development
Source: Theranostics. 2023 Mar 27;13(7):2072–87. doi: 10.7150/thno.81198 (PMC10157744; doi:10.7150/thno.81198)
Supplement: Supplementary file 1 — Supplementary figures. [file thnov13p2072s1.pdf]

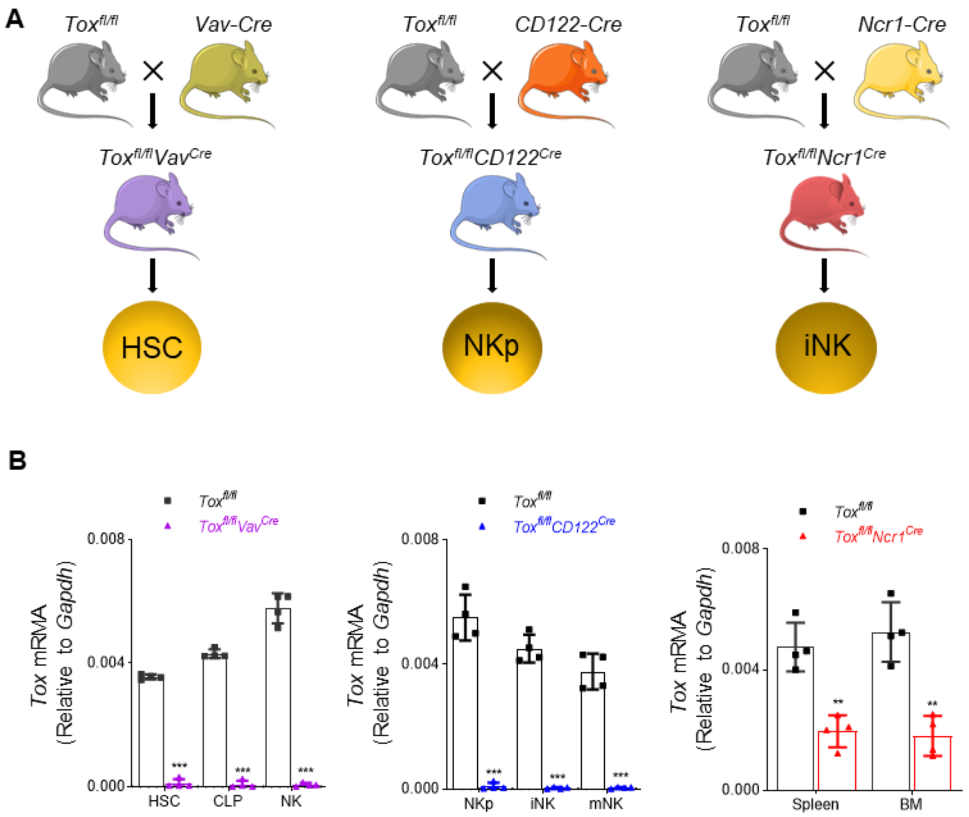


**Figure S1. Detecting TOX deletion efficiency in NK cells from the three conditional targeting mouse models. (A)** Schematic graphs showing the breeding strategies for the three conditional knockout mice. **(B)** Analysis of *Tox* mRNA expression in HSC, CLP and NK cells from BM of $Tox^{fl/fl}$ and $Tox^{fl/fl}Vav^{Cre}$ mice (left), in NKp, iNK and mNK cells from spleen of $Tox^{fl/fl}$ and $Tox^{fl/fl}CD122^{Cre}$ mice (middle), in NK cells from spleen and BM of the $Tox^{fl/fl}$ and $Tox^{fl/fl}Ncr1^{Cre}$ mice (right) by quantitative PCR. All above results were normalized to *Gapdh* (n = 4).

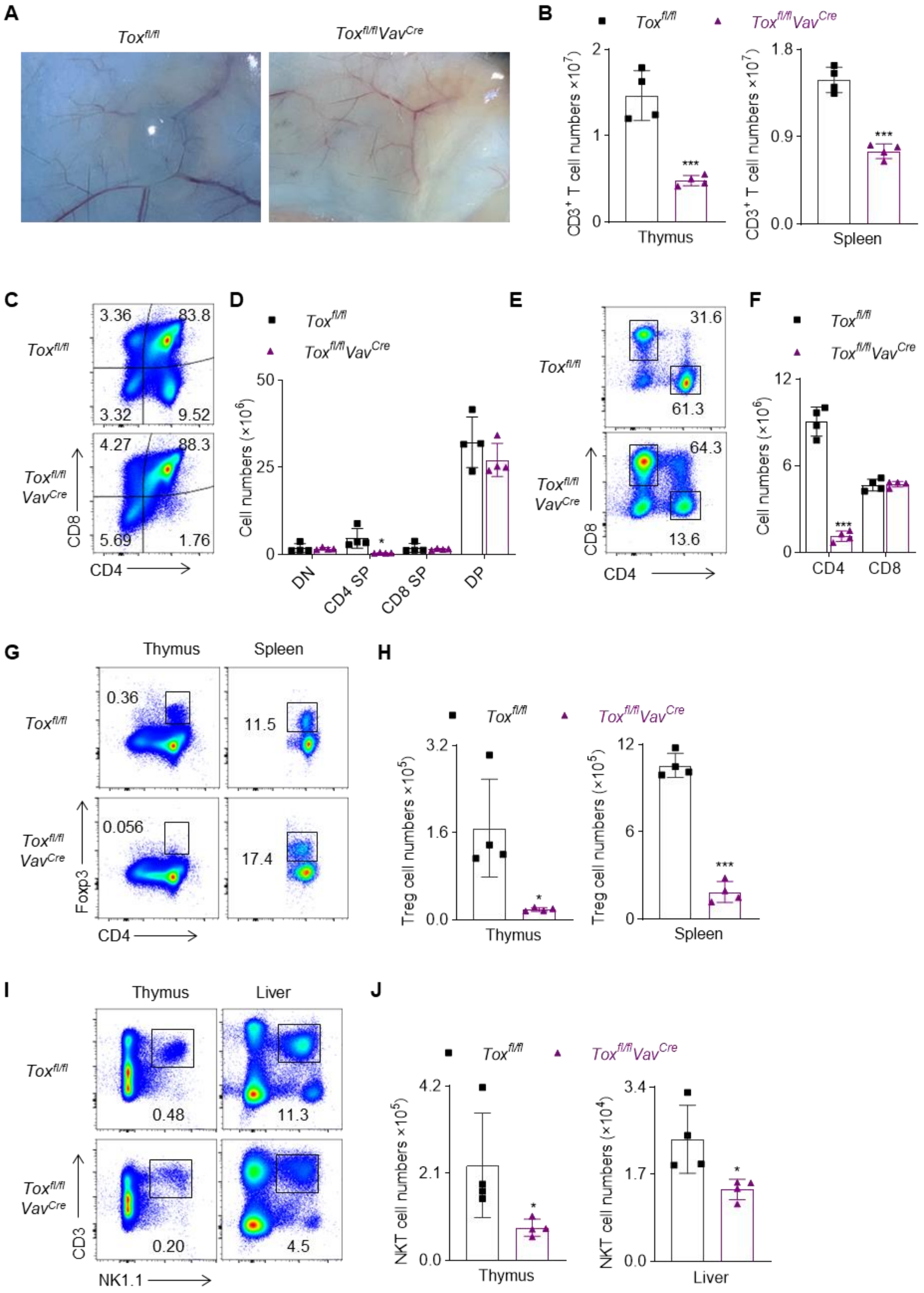


**Figure S2. The deletion of TOX at HSC stage impairs the development of CD4⁺ T cells. (A)** Gross anatomy of lymph nodes visualized by the injection of Chicago sky

blue dye into *Tox*$^{fl/fl}$ and *Tox*$^{fl/fl}$*Vav*$^{Cre}$ mice. **(B)** The number of $CD3^+$ T cells in thymus and spleen from *Tox*$^{fl/fl}$ and *Tox*$^{fl/fl}$*Vav*$^{Cre}$ mice (n = 4). **(C, D)** Representative flow cytometry plot **(C)** and the number **(D)** of different thymocyte T cell subsets in *Tox*$^{fl/fl}$ and *Tox*$^{fl/fl}$*Vav*$^{Cre}$ mice (n = 4). **(E, F)** Representative flow cytometry plot **(E)** and the number **(F)** of $CD4^+$ T and $CD8^+$ T cells in spleen from *Tox*$^{fl/fl}$ and *Tox*$^{fl/fl}$*Vav*$^{Cre}$ mice (n = 4). **(G, H)** Representative flow cytometry plot **(G)** and the absolute number **(H)** of Treg cells in thymus and spleen of *Tox*$^{fl/fl}$ and *Tox*$^{fl/fl}$*Vav*$^{Cre}$ mice (n = 4). **(I, J)** Representative flow cytometry plot **(I)** and the absolute number **(J)** of NKT cells in thymus and liver of *Tox*$^{fl/fl}$ and *Tox*$^{fl/fl}$*Vav*$^{Cre}$ mice (n = 4). Data are representative of three independent experiments.

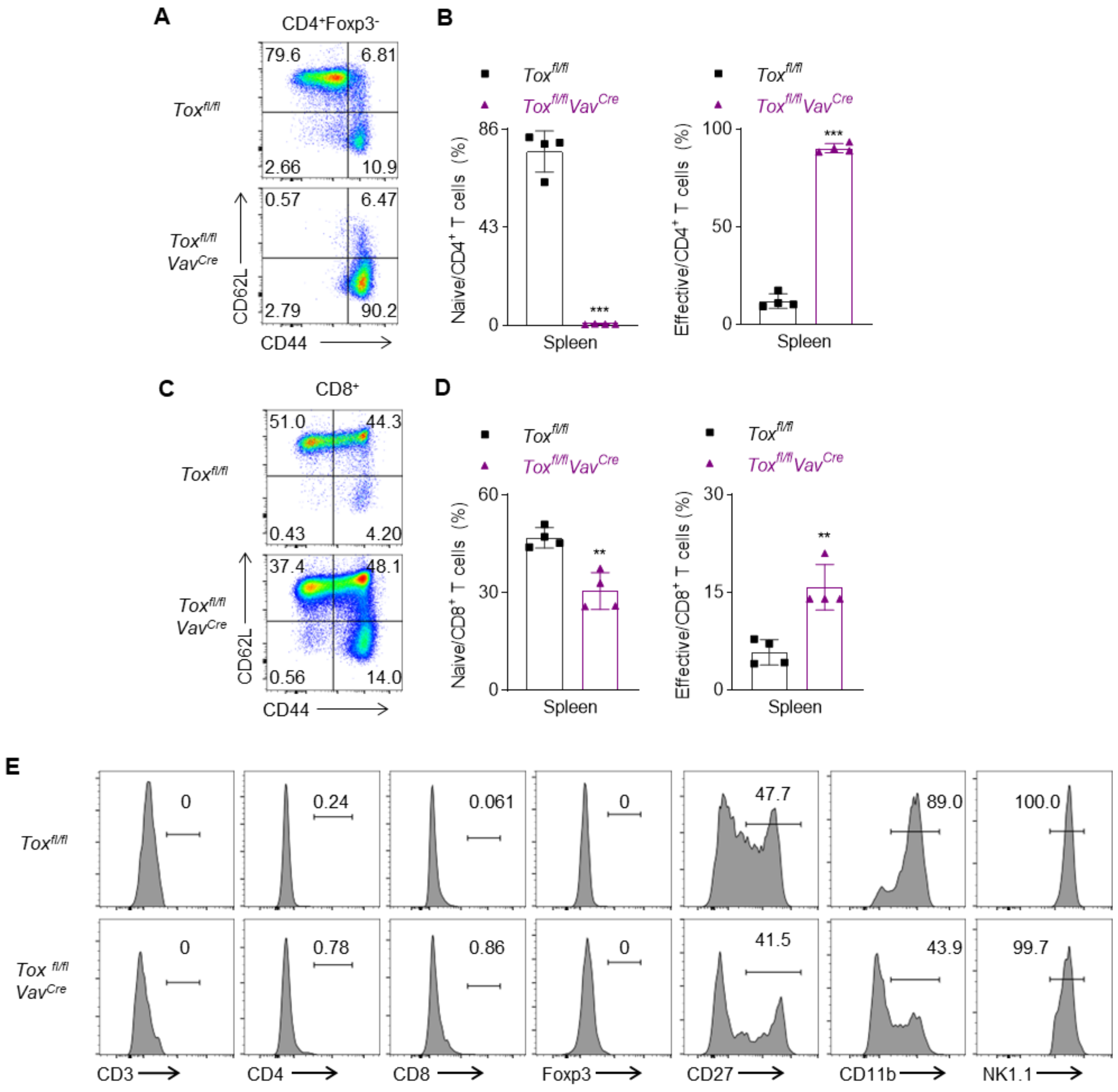


**Figure S3. Deletion of TOX at HSC stage influences the activated status of T cells.** **(A-D)** Representative flow cytometry plot **(A, C)** and the percentage **(B, D)** of naïve T cells and effective CD4$^{+}$ **(A, B)** or CD8$^{+}$ **(C, D)** T cells in spleen of *Tox$^{fl/fl}$* and *Tox$^{fl/fl}$Vav$^{Cre}$* mice (n = 4). **(E)** Comparison of the expression level of CD3, CD4, CD8, Foxp3, CD27, CD11b and NK1.1 in NK cells from spleen of *Tox$^{fl/fl}$* and *Tox$^{fl/fl}$Vav$^{Cre}$* mice. Data are representative of at least two independent experiments.

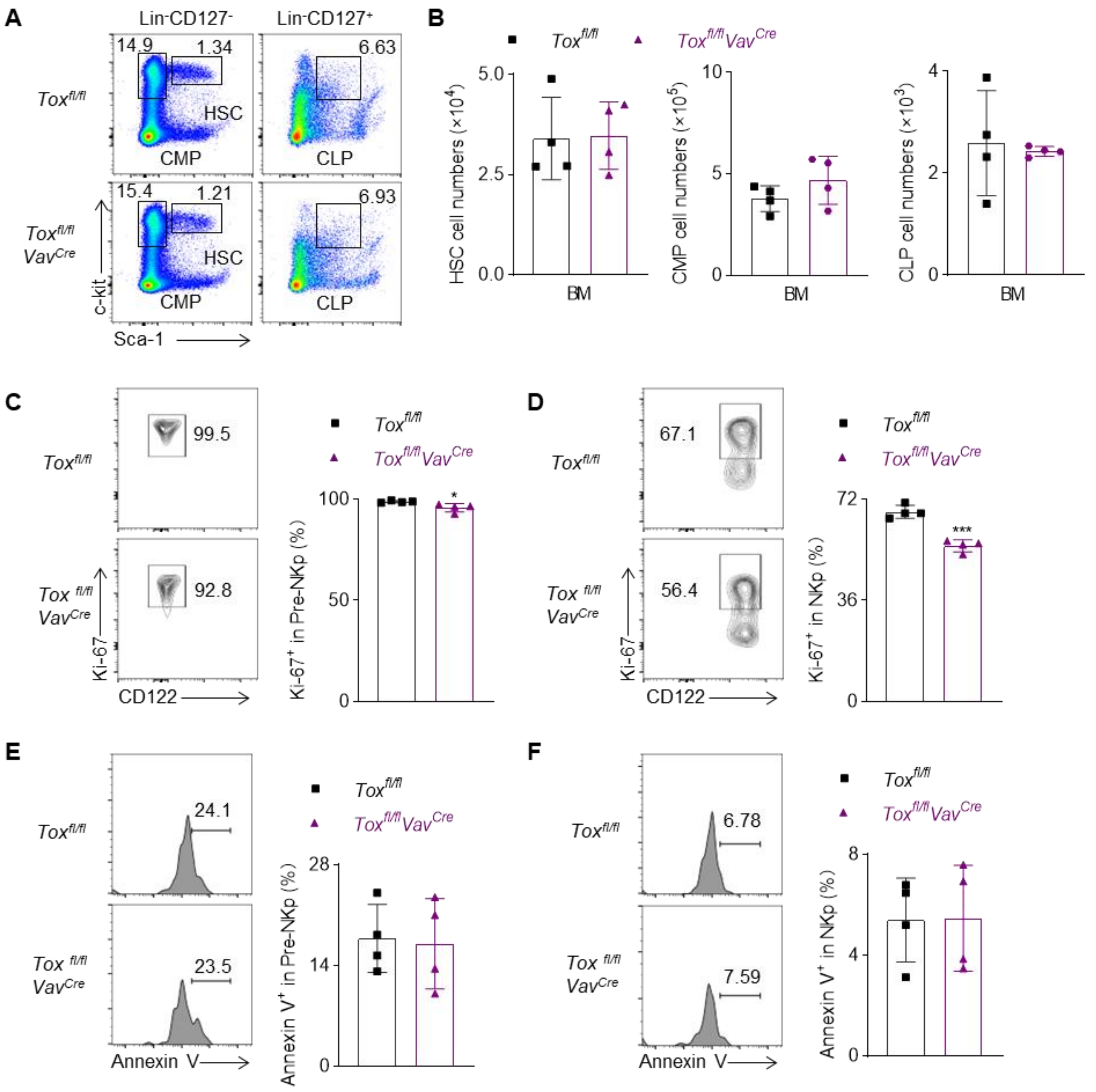


**Figure S4. TOX deletion at NKp stage impairs the proliferation capacities of Pre-NKp and NKp cells. (A, B)** Representative flow cytometry plot **(A)** and the absolute numbers **(B)** of HSC, CMP, and CLP in BM of *Tox*$^{fl/fl}$ and *Tox*$^{fl/fl}$*Vav*$^{Cre}$ mice (n = 4). **(C, D)** Comparison the expression of Ki-67 in pre-NKp cells **(C)** and NKp cells **(D)** between *Tox*$^{fl/fl}$*Vav*$^{Cre}$ mice and *Tox*$^{fl/fl}$ mice (n = 4). **(E, F)** Comparison the expression of Annexin V in pre-NKp cells **(E)** and NKp cells **(F)** between *Tox*$^{fl/fl}$*Vav*$^{Cre}$ mice and *Tox*$^{fl/fl}$ mice (n = 4). Data are representative of two independent experiments.

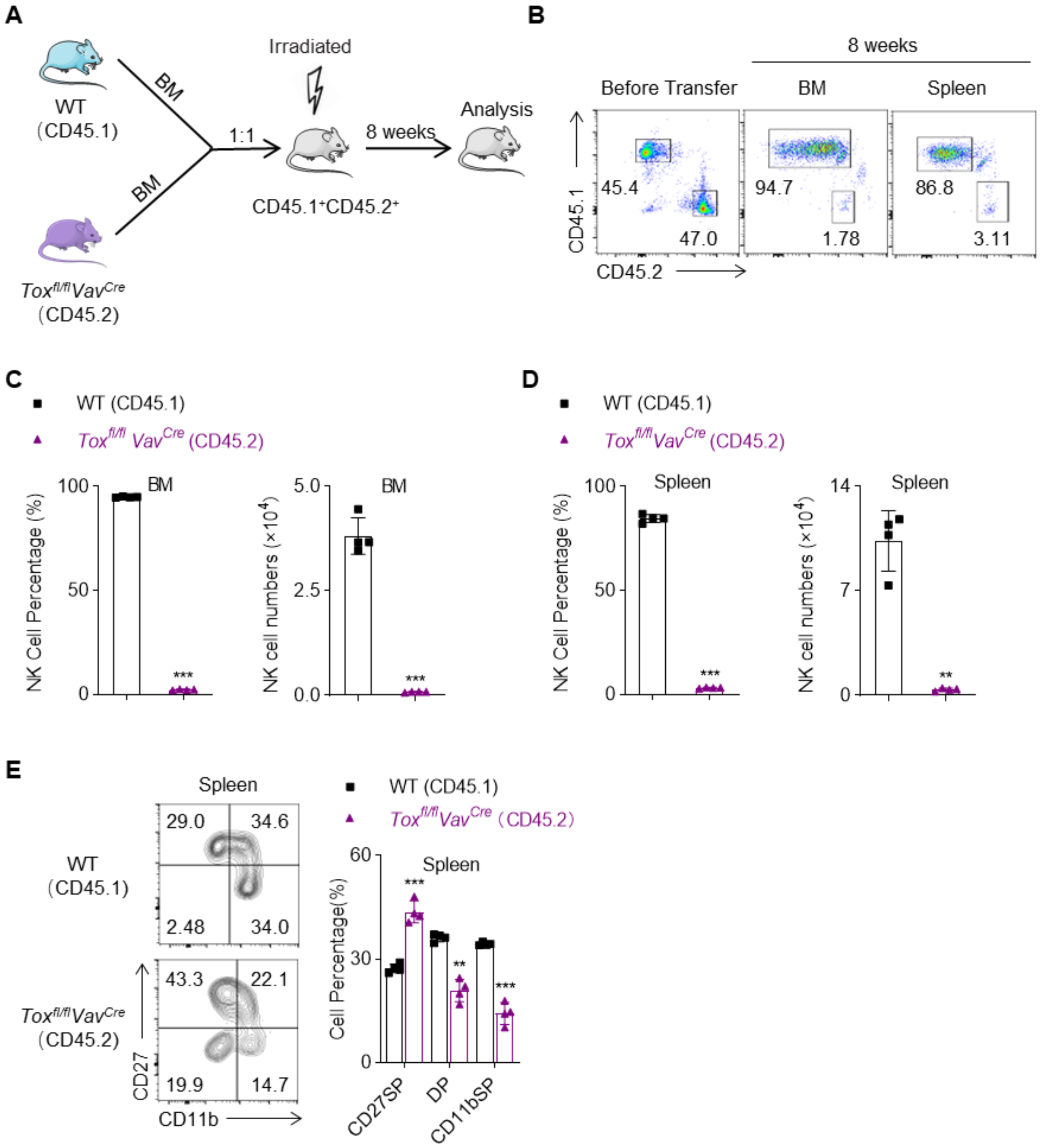


**Figure S5. TOX participates in NK cell commitment in a cell-intrinsic manner at HSC stage. (A)** Experimental design of congenic BM chimera assay. **(B)** Representative flow cytometry plots showing the percentage of NK cells from *Tox*$^{fl/fl}$ *Vav*$^{Cre}$ mice (CD45.2$^{+}$) and WT mice (CD45.1$^{+}$) in spleen and BM of recipient mice 8 weeks after congenic BM chimera assay. The injected cells are shown. **(C, D)** The percentage (left) and the number (right) of NK cells from *Tox*$^{fl/fl}$ *Vav*$^{Cre}$ mice (CD45.2$^{+}$) and WT mice (CD45.1$^{+}$) in BM **(C)** or spleen **(D)** of recipient mice 8 weeks after congenic BM chimera assay (n = 4). **(E)** Representative histogram and NK cell subset distribution from *Tox*$^{fl/fl}$ *Vav*$^{Cre}$ mice (CD45.2$^{+}$) and WT mice (CD45.1$^{+}$) in spleen

of recipient mice 8 weeks after congenic BM chimera assay (n = 4).

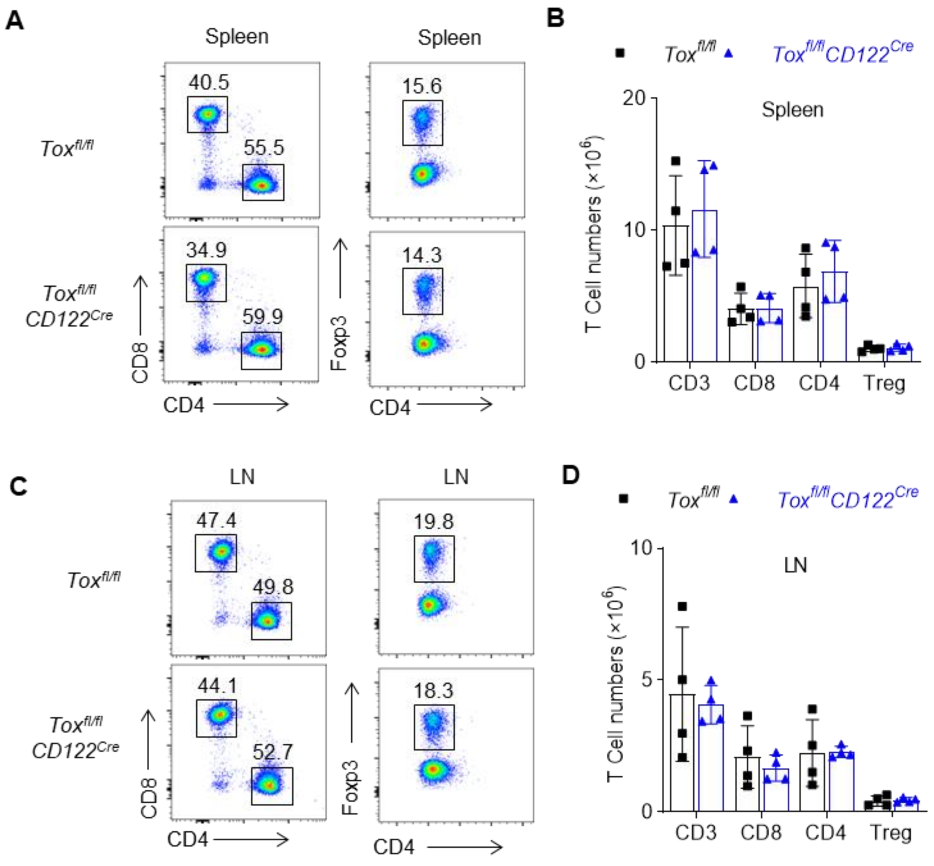


**Figure S6. The ablation of TOX at NKp stage does't affect T cell development.** Representative flow cytometry plot **(A, C)** and the absolute numbers **(B, D)** of $CD3^+$ T cells, $CD8^+$ T cells, $CD4^+$ T cells and Treg cells in spleen **(A, B)** or LN **(C, D)** of *Tox$^{fl/fl}$* and *Tox$^{fl/fl}$CD122$^{Cre}$* mice (n = 4). Data are representative of two independent experiments.

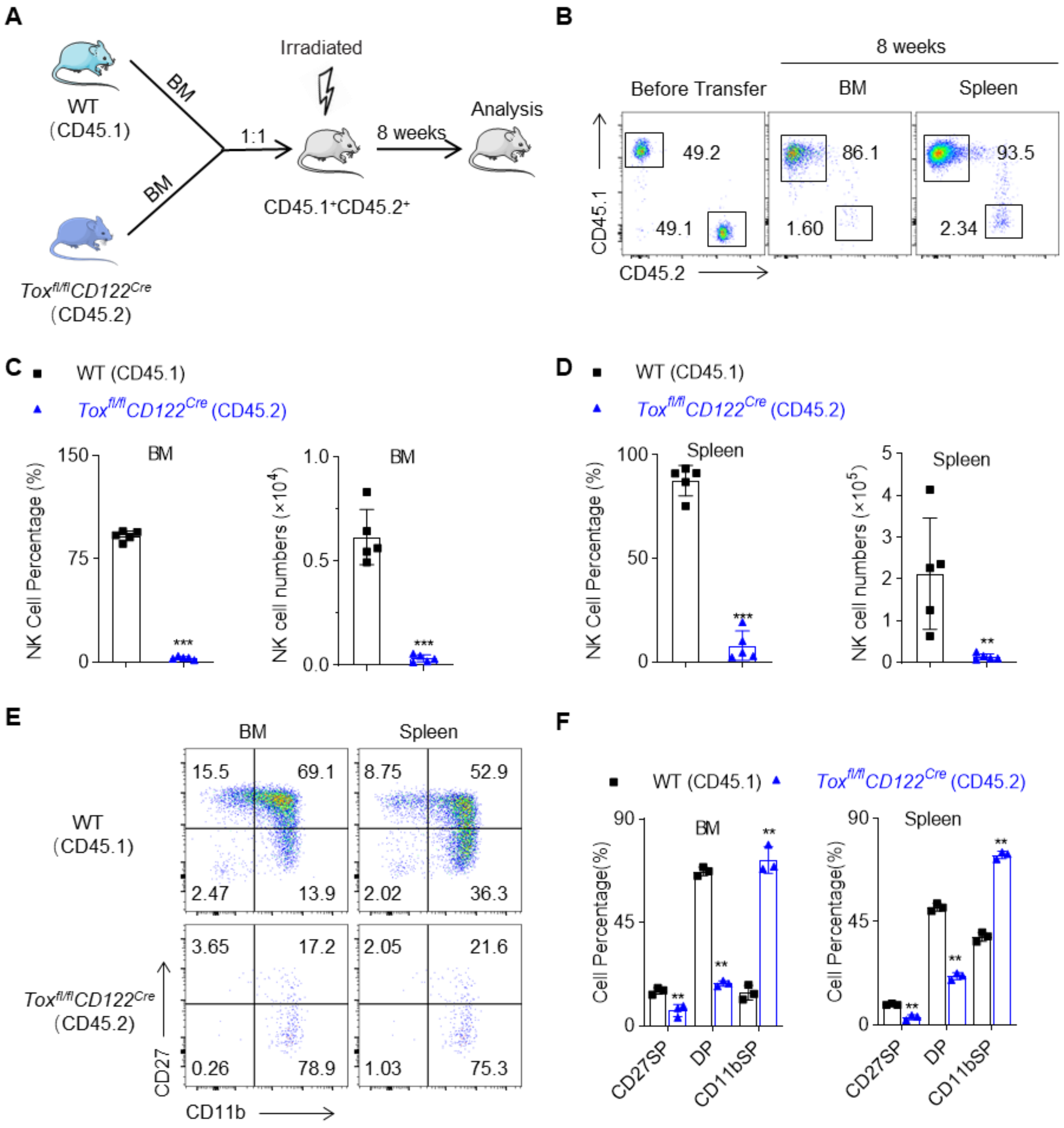


**Figure S7. Genetic ablation TOX at NKp stage leading to severe defects in NK cell development is cell-intrinsic. (A)** Experimental design of congenic BM chimera assay. **(B)** Representative flow cytometry plot showing the percentage of NK cells from *Tox^fl/fl^CD122^Cre^* mice (CD45.2$^+$) and WT mice (CD45.1$^+$) in spleen and BM of recipient mice 8 weeks after congenic BM chimera assay. The injected cells are shown. **(C, D)** The percentage (left) and the number (right) of NK cells from *Tox$^{fl/fl}$CD122$^{Cre}$* mice (CD45.2$^+$) and WT mice (CD45.1$^+$) in BM **(C)** or spleen **(D)** of recipient mice 8 weeks after congenic BM chimera assay (n = 5). **(E, F)** Representative flow cytometry plot **(E)** and the analysis of NK cell subset distribution **(F)** from *Tox$^{fl/fl}$CD122$^{Cre}$* mice (CD45.2$^+$) and WT mice (CD45.1$^+$) in BM and spleen of recipient mice 8 weeks after congenic

BM chimera assay (n = 3).

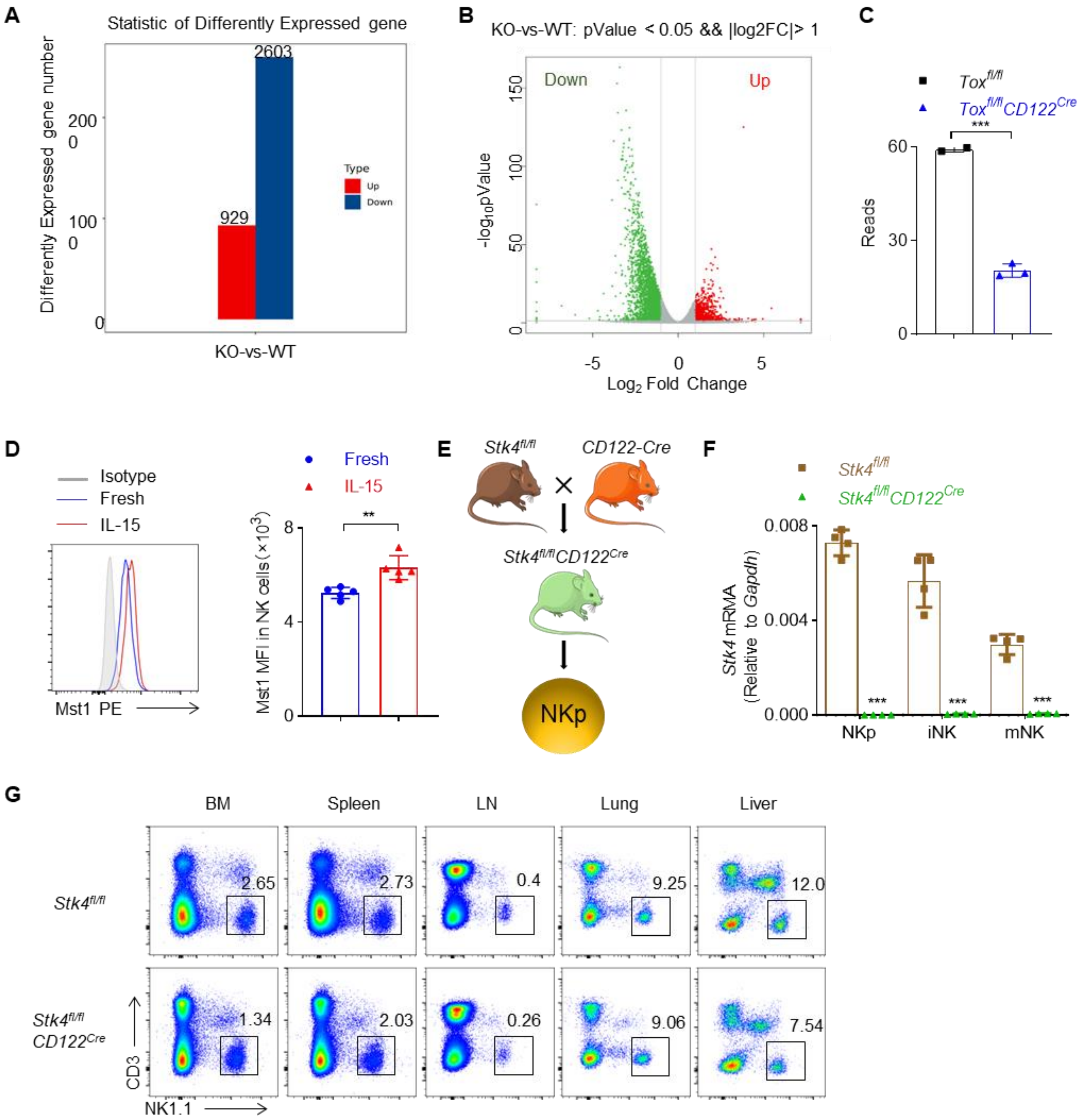


**Figure S8. TOX orchestrates NK cell homeostasis by controlling Mst1 expression. (A, B)** The histogram **(A)** and the volcano plot **(B)** showing differentially expressed genes from RNA-seq. **(C)** The expression level of *Stk4* between WT NK cells and TOX-deficient NK cells in our RNA-seq dataset. **(D)** Comparison the expression level of Mst1 in NK cells from spleen of WT mice before and after stimulation with 10 ng/mL IL-15 for 16 h (n = 4)**. (E)** Schematic graph showing the strategy for generating the *Stk4*$^{fl/fl}$*CD122*$^{Cre}$ mice. **(F)** Analysis of *Stk4* mRNA expression in NKp, iNK and mNK

cells in spleen of *Stk4*$^{fl/fl}$ and *Stk4*$^{fl/fl}$*CD122*$^{Cre}$ mice by quantitative PCR. **(G)** Representative flow cytometry plots showing the percentage of NK cells in BM, spleen, LN, lung and liver of *Stk4*$^{fl/fl}$ and *Stk4*$^{fl/fl}$*CD122*$^{Cre}$ mice.
